# *De novo* assembly of *Agave sisalana* transcriptome in response to drought stress provides insight into the tolerance mechanisms

**DOI:** 10.1038/s41598-018-35891-6

**Published:** 2019-01-23

**Authors:** Muhammad Bilal Sarwar, Zarnab Ahmad, Bushra Rashid, Sameera Hassan, Per L. Gregersen, Maria De la O. Leyva, Istvan Nagy, Torben Asp, Tayyab Husnain

**Affiliations:** 1 Plant Genomics Lab, Center of Excellence in Molecular Biology, University of the Punjab, 87-West Canal Bank Road Thokar Niaz Baig, Lahore, 53700 Pakistan; 2 Department of Molecular Biology and Genetics, Aarhus University, Forsøgsvej 1, Slagelse, Denmark

## Abstract

Agave, monocotyledonous succulent plants, is endemic to arid regions of North America, exhibiting exceptional tolerance to their xeric environments. They employ various strategies to overcome environmental constraints, such as crassulacean acid metabolism, wax depositions, and protective leaf morphology. Genomic resources of Agave species have received little attention irrespective of their cultural, economic and ecological importance, which so far prevented the understanding of the molecular bases underlying their adaptations to the arid environment. In this study, we aimed to elucidate molecular mechanism(s) using transcriptome sequencing of *A. sisalana*. A *de novo* approach was applied to assemble paired-end reads. The expression study unveiled 3,095 differentially expressed unigenes between well-irrigated and drought-stressed leaf samples. Gene ontology and KEGG analysis specified a significant number of abiotic stress responsive genes and pathways involved in processes like hormonal responses, antioxidant activity, response to stress stimuli, wax biosynthesis, and ROS metabolism. We also identified transcripts belonging to several families harboring important drought-responsive genes. Our study provides the first insight into the genomic structure of *A. sisalana* underlying adaptations to drought stress, thus providing diverse genetic resources for drought tolerance breeding research.

## Introduction

Drought is one of the major abiotic stresses, which significantly diminishes the agricultural production and threatens food security worldwide^1^. Sessile nature of plants limit them to their natural habitat, therefore many species have evolved appropriate mechanisms to cope with the drought stress such as drought escape, avoidance, and tolerance that may act synergistically^2^. The employed mechanism largely depends on multiple factors e.g plant species, developmental phase, duration and severity of the drought progression^3^. All these adaptive mechanisms are complex, polygenic in nature, requiring, physio-biochemical and molecular changes in order to survive^4^. These changes involve a number of drought particular transcripts that can be associated with two broad groups; “functional proteins” versus “regulatory proteins”^5^. The induction and accumulation of the functional proteins include dehydrins, photosynthesis-related genes, aquaporins, lipid transfer proteins, biosynthesis and transport of various osmoprotectants, protein repair enzymes, proteases, protease inhibitors, and other enzymes, directly guard the cells against the abiotic factors^5,6^. The regulatory proteins largely involved in the immediate response to drought stress by directing the expression of downstream genes. These proteins include transcription factors (TFs), protein kinases and phosphatases encoding genes, genes involved in the biosynthesis of abscisic acid (ABA) that control the stomatal behavior and other physiological phenomena^5,7^.

The Agave is predominantly monocarpic, succulent, xerophytic plants belonging to Asparagaceae family. This genus comprehends more than 166 species, native to the arid and semi-arid origin of Mexico^8^. Presently they are grown in almost every agricultural area of the world because of their extreme ecological adaptation^9^. In Pakistan, Agave is represented by six cultivated species^10^. Many of agave species are of great commercial importance for their use in food, fiber, shelter, insecticides, and ornamentals^11^. *Agave tequilana* usually known as “blue agave” is useful to prepare alcoholic beverages such as “pulque” and “tequila” which earns $1.7 billion per annum within the United States^12^. “Sisal” is the sixth most important fiber, harvested from the *Agave sisalana* Perr. ex. Engelm, representing 2% of the world’s production of plant fibers^13^. *A. sisalana* is a hardy plant that displays exceptional drought and temperature tolerance. It grows well all year round in hot and extremely dry climate^14^.

The leaves and stem of the agave is the rich source of carbohydrates and lignocelluloses and introduced as a lingo-cellulosic bioenergy feedstock. Its average yield falls in the range of 8.5 to 22 Mg ha^−1^ yr^−1^ of dry weight under mild climate conditions^15,16^. Persistent aridity, with no relief of irrigation, harshly damage the yield to 2.0–5.0 Mg ha^−1^ yr^−1^ dry mass. However, an adequate level of management and resource input may lead to 38 and 42 Mg ha^−1^ yr^−1^ yield for some species^17^. Its use for bioenergy production could result in higher yield than other energy crops, such Zea mays (15–19 Mg ha^−1^), miscanthus species (29–38 Mg ha^−1^), and *Panicum virgatum* (10–12 Mg ha^−1^)^18^.

Remarkable tolerance to abiotic stresses makes the Agave species an ideal plant to explore essential genomic information for abiotic stress traits. The crassulacean acid metabolism (CAM) mechanism makes them possible to utilize the water 4−2x more efficiently^12^. They have the inbuilt ability to survive more than one season without rainfall and can tolerate extremely hot and low temperatures (−16.1 °C to 61.4 °C)^19^. Agave has the large, complex genome, estimated between 2940 to 4704 Mbp of DNA in size with a high level of duplication due to polyploidy levels (2x, 3x, 4x, 5x, 6x, and 8x)^20^. Notwithstanding, its economic and ecological potential towards the abiotic stress, a limited investigation has been carried out yet. There is just a single transcriptome base *de novo* assembly reported for species *A. tequilana* and *A. deserti*^12^. Therefore, further comprehensive genome-scale studies are lacking to explore out the molecular basis for adaptation of agave to harsh conditions. Whole transcriptome analysis using the Next-generation sequencing (NGS) enables us to understand the expression patterns in response to the environmental stress. In parallel, advancements in computational tools overcome the complication that may arise due to the lack of suitable well-annotated reference genome for non-model plant species^21^. These tools assemble the raw reads into short DNA *de novo* sequences, “contigs”, which enables various downstream analyses like gene discovery, mutation detection, and expression analysis. Transcriptomes of non-model organisms via *de novo* assembly has been reported for numerous plants^12,22–25^.

In this study, we aim to fill the gap in the existing knowledge on the transcriptional response by the agaves to the drought stress. A *de novo* assembly of Illumina platform generated reads was carried out to provide a thorough scenario on the *A. sisalana* transcriptome under drought stress. The study of differential gene expression and their possible pathways analysis should improve the current knowledge to understand the molecular basis behind the adaptation and survival of agaves in a xeric environment. The present work not only enriches the available knowledge about the genome of agave species but also provides an important transcriptomic database for further molecular investigation.

## Results

### RNA-Seq data overview

To explore the drought tolerance mechanism(s) at the molecular level, we sequenced and analyzed the leaf specific transcriptome of *A. sisalana* by mRNA sequencing. Six paired-end cDNA libraries were generated from three well irrigated (control: C1, C2, C3) and from three droughts stressed (drought: T1, T2, T3) independent biological samples. The Illumina sequencing platform Hiseq2500 was used for paired-end sequencing at Macrogen Korea with the insert size 101 bp. A total of 276,845,790 reads and 27,961,424,790 nucleotides were sequenced (Supplementary Information 1a) (Table 1). The data of individual biological library were deposited to NCBI SRA database with SRA accession IDs: SRR5137659, SRR5137661, SRR5137662, SRR5137658, SRR5137663, and SRR5137660. Supplementary Information 1b represents the complete workflow and experimental design.

**Table 1:** Numerical Summary of the Illumina generated raw reads and denovo assembly statistics.

Contents	Control Library (C)	Drought Library (T)	Total
**RNA-Sequencing Statistics**			
Number of clean reads	152553060	124292730	276845790
Total base pairs (bp)	15407859060	12553565730	27961424790
Q20 percentage (%)	97.8%	95.9%	96.85%
N Percentage	0	0	0
GC percentage	48.29%	47.7%	48.1%
**Assembly Statistics**			
		**Contigs**	**Unigene**
Total number of sequences		93141	67328
average length		731 (bp)	582 (bp)
N50		1164 (bp)	834 (bp)
Min length		201 (bp)	201 (bp)
Max length		9304 (bp)	9304 (bp)

### Transcriptome *De novo* assembly and evaluation statistics

**Figure 1:**
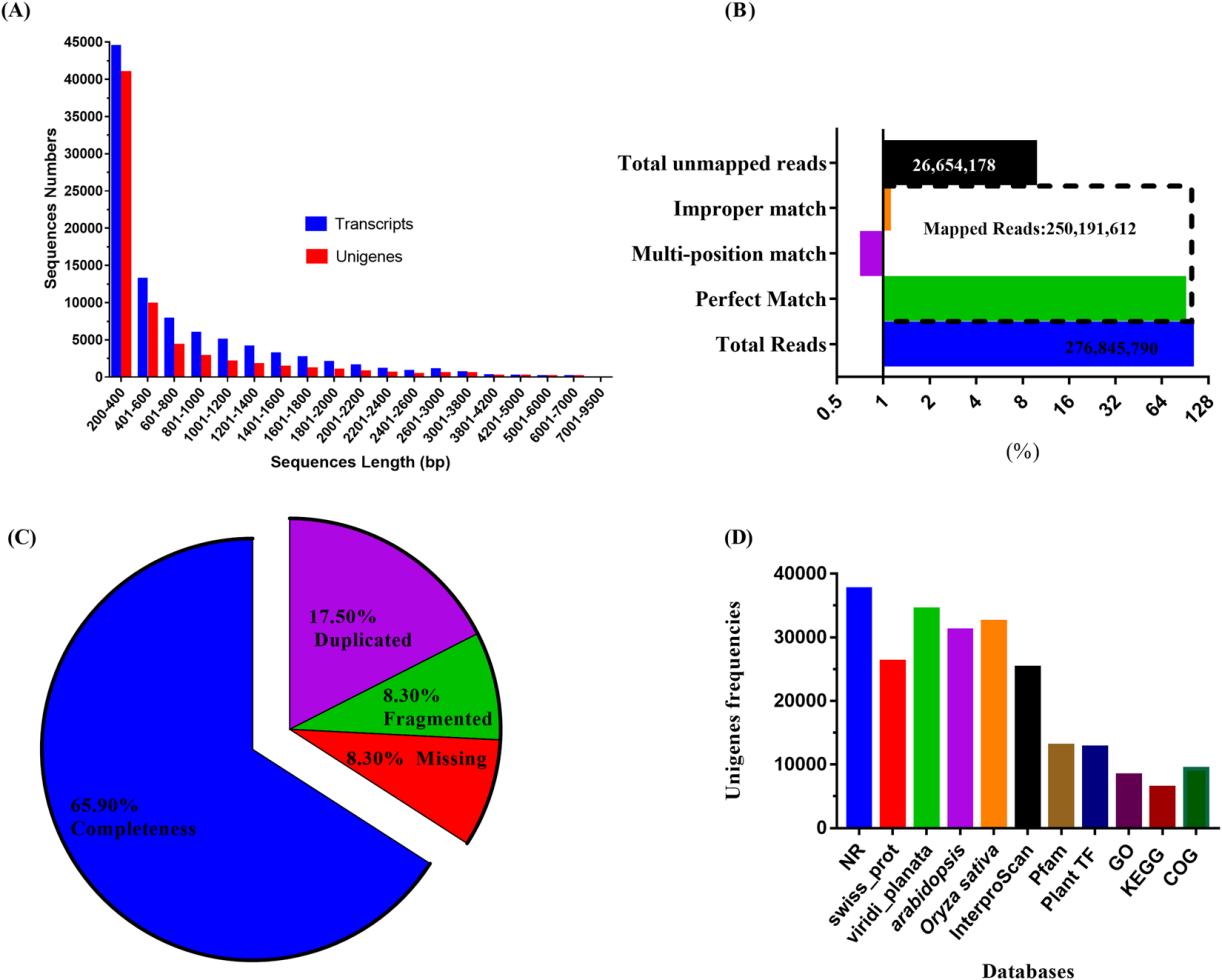
(**A**) Sequence length distribution of the transcripts and unigenes of the trinity generated *de novo* assembly driven out of the raw reads from the control and drought stress samples. (**B**) Graphical representation of the statistics of cleaned raw reads mapping back to the *de novo* assembled transcripts (RMBT). (**C**) Benchmarking Universal Single-Copy Orthologs (BUSCO) scores for assembly quality assessment. (**D**) Homology analysis of the non-redundant unigenes against the publically available databases.

*De novo* assembly is an efficient and comprehensive way for the discovery of novel transcripts, their expression behavior and new markers in the absence of the whole genome sequencing data. Length distribution pattern of transcripts produced by assemblers (see materials and methods) was generated against the well-annotated *Ananas comosus* CDS (https://phytozome.jgi.doe.gov) and transcriptome based *A. deserti* reference sequences (http://datadryad.org/). Trinity generated assembly correlates closest to the reference’s distribution followed by the Trans-ABySS (64 K-mer) and Short Oligonucleotide Analysis Package (SOAP) (Supplementary Dataset 1 S1, S2). The results may vary from one dataset to others, and so the user should optimize their own preferences/settings according to the data type. We have successfully assembled the 276.8 million reads with Trinity into 93,141 contigs (transcripts hereafter) and 67,328 longest isoforms per gene (unigene hereafter) with 68,048,194 and 39,203,184 bp nucleotides in counts respectively (Supplementary Dataset 1-S3) Table 1. The transcripts and unigenes were in-between 200–9304 bp by length span, with an average length of 731 bp and 582 bp respectively. On average, there were about 43,396 transcripts in the range of 200–400 bp, 26,728 in 401–1000 bp, 16,536 in 1001–2000 bp, 4736 in 2001–3800 bp and 398 transcripts hold >4000 bp, while this counts for unigene were 40849, 16788, 6977, 2461 and 253 respectively (Fig. 1A). GC percentage content (45.3%) of *A. sisalana* assembly was quite similar to the *A. deserti* (45.1%) than *A. tequliana* (42.3%) and *O. sativa* (55%). Further, a Perl supported script orfPerdictor predicts 92,559 (99.3%) and 63,589 (94.3%) sequences having potential readable ORF from the transcripts and unigenes data, respectively. Additionally, BLAST analysis (Blastp with e value 1e^−20^) against the viridiplantae (Taxon_ID 33090) database returned more than 25000 sequences with significant hits for both queries (contings and unigenes) (Supplementary Dataset 1-S4). The transcriptome completeness and the quality of *de novo* assembled reference are critical for the accuracy of the downstream analysis like gene identification, differential gene expression analysis, and genetic molecular developments. RMBT and BUSCO V2 16 are the most widely used packages for the assessment of the *de novo* assembly. Several recent studies have used the BUSCO tool, as the results have been demonstrated to be more solid than the previously used packages like CEGMA (Core Eukaryotic Genes Mapping Approach) and N50 statistics^26^. Almost 95% of the reads were mapped back to transcriptome by bowtie2 (RMBT) with 83% completeness while duplication percentage was ~21% as by BUSCO analysis (Fig. 1B,C). This intermediate to high duplication level may be due to the higher polyploidy level of the Agave genome. All these indicators supported that we have generated a high-quality transcriptome assembly that could be used for further possible downstream analysis.

### Functional characterization of the assembled transcriptome

**Figure 2:**
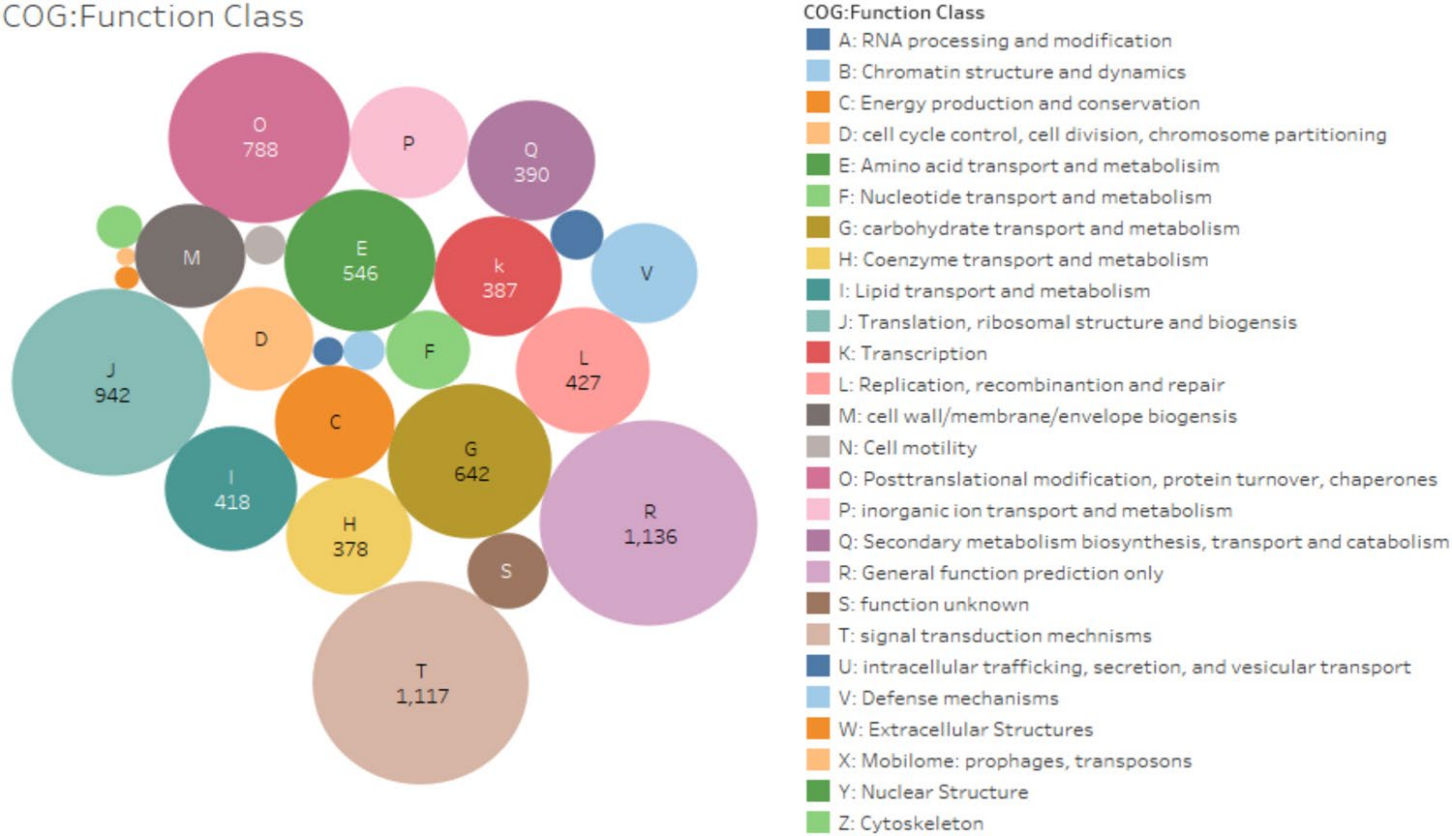
Clusters of orthologous group-based classification of all unigenes. The unigenes were aligned to the COG database (1e^−5^) to understand their possible protein function. In total 9307 unigenes were annotated and grouped into the 26 categories. The capital letter indicates the COG categories listed on the right while numeric represents the total number of unigenes in each category.

**Figure 3:**
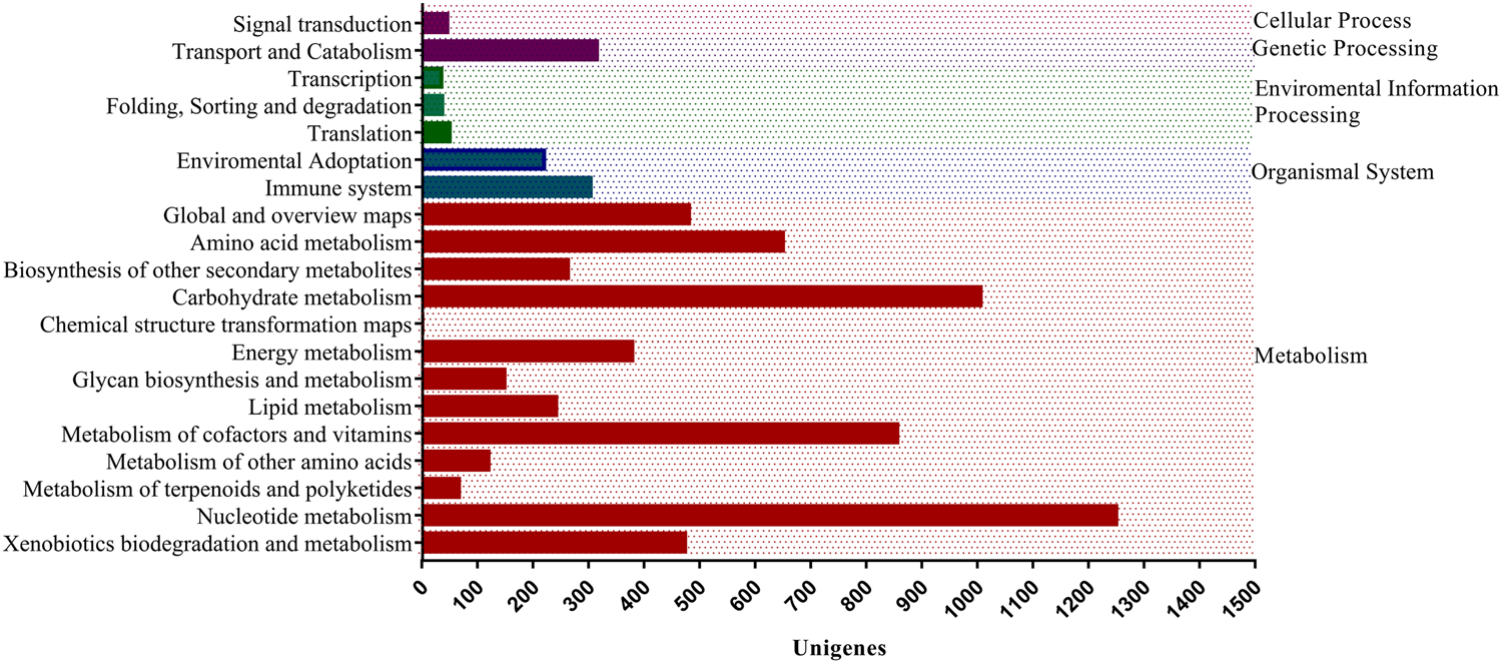
Pathways classification into metabolism, organismal system, environmental information processing, cellular process and genetic processing major groups based on the KEGG analysis.

**Figure 4:**
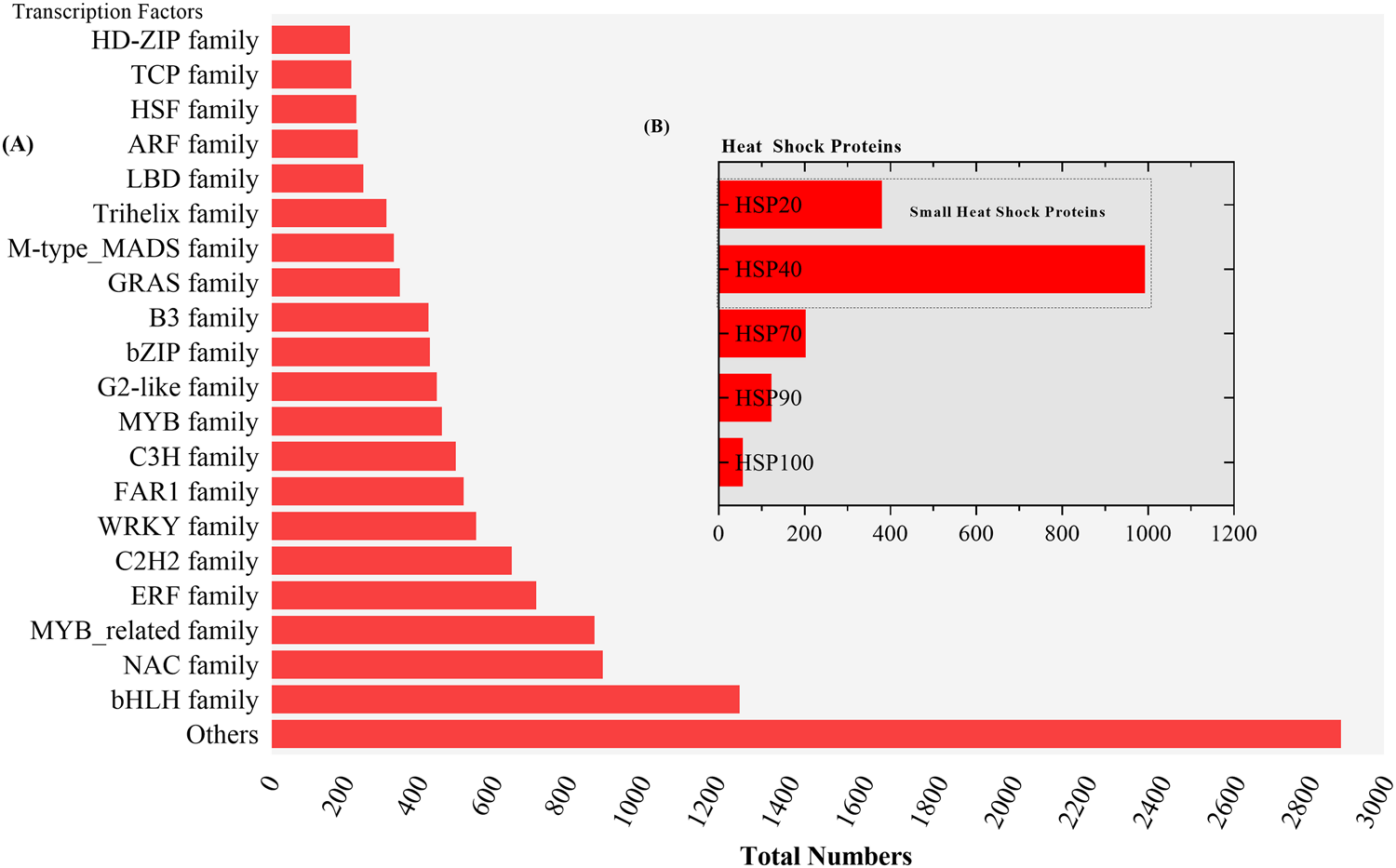
Total genes occupied a proportion of the (**A**) transcription factors and (**B**) heat shock proteins families in the *A. sisalana de novo* assembled transcriptome.

The assembled *A. sisalana* transcriptome features and functional annotations were based on top hits mapping information from nr database (1.0 e^−5^), then viridiplantae (51.3%), UniProt (38.6%), *Arabidopsis thaliana* (46.2%), *O. sativa* (48.2%), Pfam (37.5%), Gene Ontology (GO) (24.01%), PlantTF database(1e^−10^) (18.8%) and Cluster of Orthologous Groups of proteins (COG) (13.8%) Fig. 1D (Supplementary Dataset 1-S5). In total 37,546 unigenes assigned functions out of the 67,328 (E-value ≤ 1e^−5^), which may be due to fewer homologous sequences of *A. sisalana* in the public database. Maximum homology with sequences from the species like *Elaeis guineensis* (31%), *Phoenix dactylifera* (27%), and *Musa acuminata* subsp. Malaccensis (9%) and others were obtained by BLAST search. This similarity index reflects the close genetic relationship with these species (Supplementary Dataset 1-S6a). Though the leaf sampling was performed in the greenhouse from clean tissues, interestingly, we also got hits outside plants domian like Metazoa, bacteria, fungi, and Amoebozoa, etc. (Supplementary Dataset 1-S6b). Further, GeneMARK (http://exon.gatech.edu/GeneMark/) a standalone gene prediction package retrieved 24,797 functional unigenes having minimum 98 amino acid residues. The unpredicted may have less amino acid residues than the predicted or could be the assembler misassembles or novel sequences. The 9307 (14%) unigenes were divided into 25 categories for functional prediction and classification matching the Cluster of Orthologous Group (COG database; e value 1e^−5^) (Fig. 2). As per GOSlim distribution, most transcripts were related to the biological process (BP) 58.2%, then molecular functions (MF) 43.2% and cellular components (CC) 35.7%. (Supplementary Dataset 2 S1). We obtained 129 biochemical pathways with the involvement of 6338 unigenes based on KEGG database prediction (http://genome.jp/kegg/) under drought stress (Supplementary Dataset 2 S2, S3). These unigenes were further categorized into five diverse functional groups, namely metabolism (93.4%), the organismal system (4.5%), environmental information processing (0.72%), genetic information processing and cellular processes (0.78%) (Fig. 3). The diverse metabolism category had 5876 unigenes, most of which were involved in nucleotide metabolism (21.05%), carbohydrate metabolism (16.9%), metabolism of cofactor and vitamins (14.4%), amino acid metabolism (10.94%), global and overview maps (8.1%) and other eight subcategories (28.54%). Purine and pyrimidine metabolism were the core group in nucleotide metabolism and treated as the housekeeping function within the plant kingdom. Evidence suggests that they involved in the stress protection to abiotic stress tolerance via activation of the ABA metabolism pathway^27^. In the biosynthesis of secondary metabolites, the most frequent subsets of sequences were Phenylpropanoid biosynthesis (37.06%), Tropane, piperidine and pyridine alkaloid biosynthesis (14.6%) and Novobiocin biosynthesis (14.3%) (Supplementary Dataset 2-S4). Transcription factors are the main upstream regulatory elements that control the gene expression of sessile nature plants through specific binding to the *cis-*regulatory elements present in the promoter regions. We predicted, 12,676 transcription factors from the unigenes database and their annotation was retrieved from the PlantTFDB. The major families were associated with the bHLH (9.93%) group, followed by the NAC family (7.02%), MYB related group (6.8%), ERF family (5.6%), C2H2 group (5.09%), WRKY (4.33%), FAR1 (4.07%), C3H (3.9%), MYB group (3.6%) (Fig. 4A). All these are considered to be involved in the regulation of metabolic and secondary metabolic biosynthesis in the green plants^22,25,28^. Heat Shock protein annotation was retrieved based on the Heat Shock Protein information resource database (http://pdslab.biochem.iisc.ernet.in/hspir/) (Fig. 4B).

### Drought responsive transcripts identification and GO tagging

**Figure 5:**
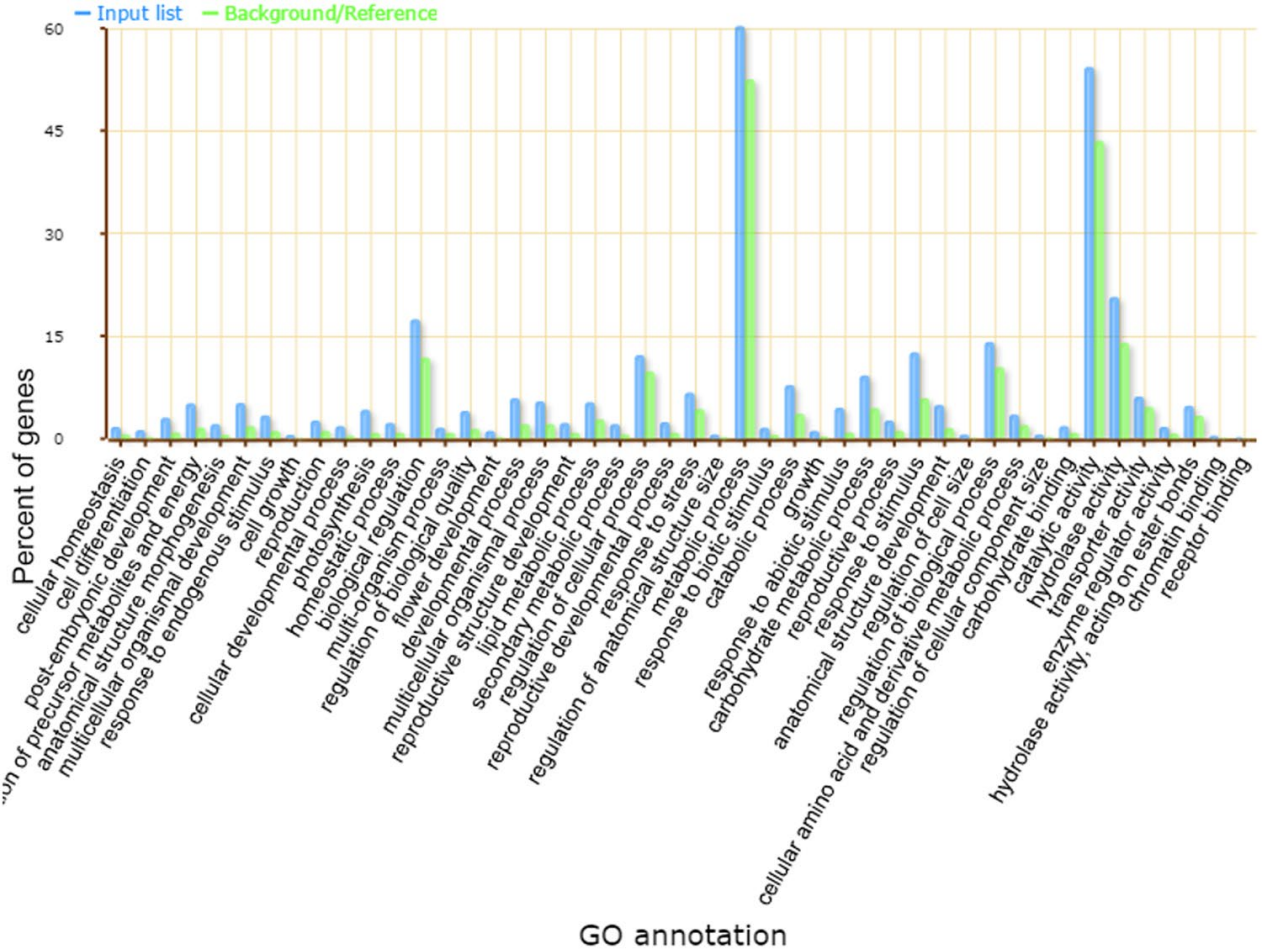
GO terms enrichment analysis of all the differentially expressed genes was performed by the AgriGO online tool. Percentage of genes that were associated with specific GO terms are shown on left side of the graph.

**Figure 6:**
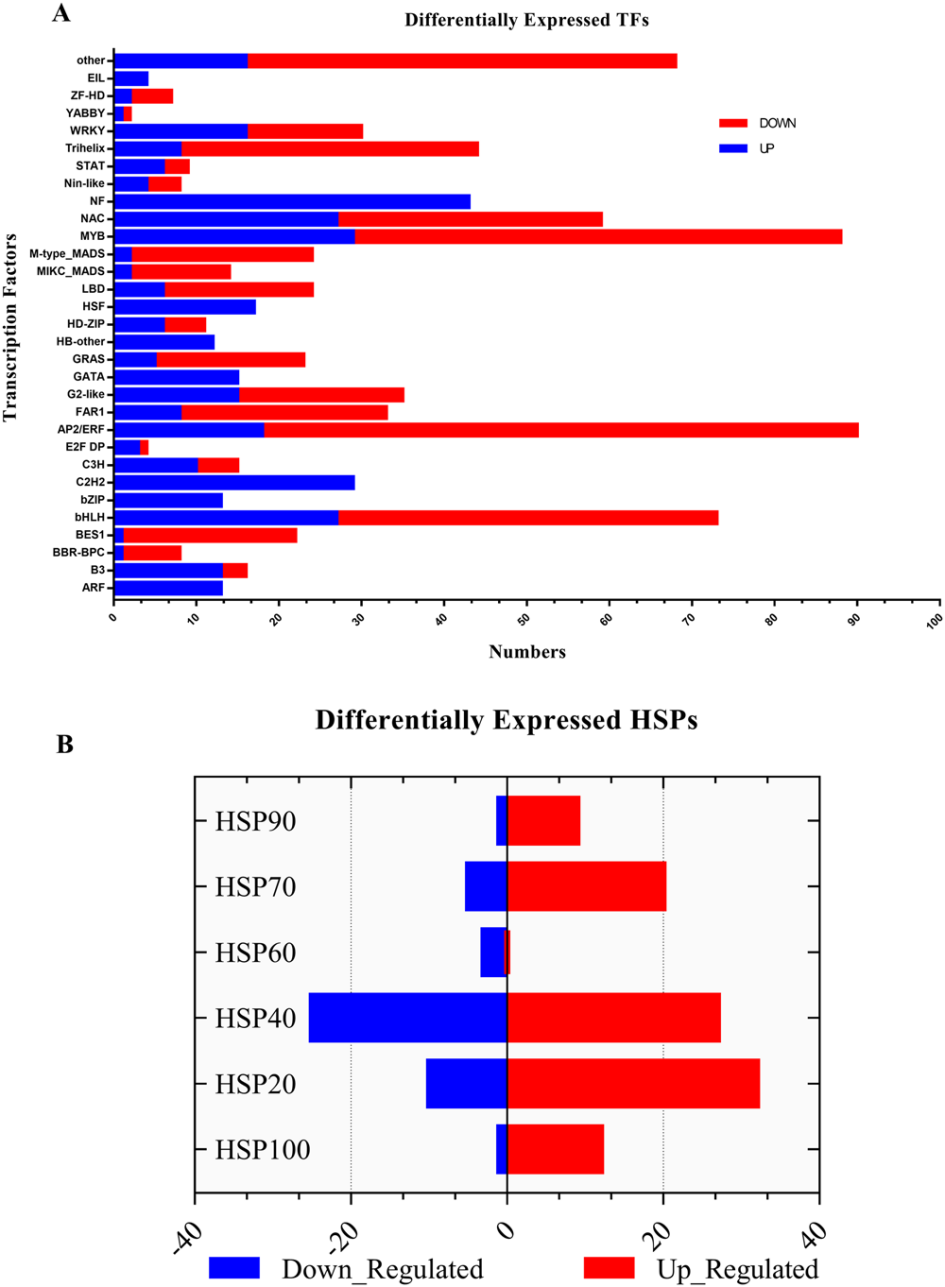
(**A**) Total number of up and down-regulated transcription factors and their response to drought stress. Within the red bar and blue colors indicating the up-regulated and down-regulated genes respectively. (**B**) Heat Shock Protein families response to the drought stress.

To investigate the differential gene expression among control and drought group, the bioconductor package edgeR was used on the read counts data that was generated by RSEM package. In total 3095 differentially expressed unigenes (DEG) significantly differed between normal and drought conditions with ≥1-fold expression (log2- fold change)| and FDR less than 0.001 confidence interval. Among these, 1195 genes were up-regulated, while 1864 were down-regulated (Supplementary Information 2 S1–S4). Out of these, 2472 (79.3%) unigenes showed homology in the nr database (2682), viridiplantae (2474), Swiss-Prot (2,047), InterPro scan (2,047), Pfam (2,047), GO (1,438), COG (819) and the KEGG (1435) (Supplementary Information 2 S1). KEGG analysis predicted the involvement of DEGs in 114 pathways. Purine metabolism pathway (ko00230;1104 unigenes) was at the top with the highest DEGs involvement, followed by Thiamine metabolism (Ko00730; unigenes 608), Biosynthesis of antibiotics (Ko0079; 481unigenes), starch and sucrose metabolism (Ko00500; 291 unigenes) and Aminobenzoate degradation (Ko00627;168 unigenes). Various significant drought specific pathways and enzymes belonging to metabolism and other groups were also discovered (Supplementary Information 2-S2). On the basis of gene ontology database, 42%, 36.5% and 29% of DEGs were assigned GO terms in the categories of Biological process, Molecular Functions, and Cellular Components respectively. Search against the COG database divided these DEG into 25 functional groups. Carbohydrates transport (16.06%), posttranslational modification (13.5%), chaperones general function prediction only (9.9%), lipid transport and metabolism (8.8%), signal transduction mechanisms (7.7%) were the most frequent categories. Enriched GO terms specific to drought stress were also identified with Singular Enrichment Analysis (SEA) at 0.05 significance interval (Fig. 5). In total 107 significantly enriched GO terms were identified, including response to abiotic stimulus (GO:0009628), photosynthesis (GO:0015979), response to stimulus (GO:0050896), binding (GO:0005488), cell communication (GO:0007154), transcription (GO:0006350), metabolic process (GO:0008152), cellular process (GO:0009987), catalytic activity (GO: 0003824) and others (Supplementary Dataset 3). The role of transcription factors in the plant response to the abiotic stress is critical and have been studied in a variety of species^29,30^. Here the GO terms for transcription (GO:0006350), transcription regulator activity (GO:0030528) and transcription factor activity (GO:0003700) were significantly enriched indicating enhanced activity under drought stress. Total 1178 DEGs were predicted as the potential TFs under drought stress in *A. sisalana* transcriptome, and were further classified into 52 subfamilies (Supplementary Dataset 4-S1). Majority of these genes belonged to ERF family (102), bHLH (100), NAC group (86), MYB_releated (84), C2H2 group (58), WRKY family (46), HSFs (33) and others (Fig. 6A). Heat shock proteins (Hsps) are classified into five major categories based on molecular mass. The differential expression of genes within these categories was calculated based on the fold change. Collectively145 differentially expressed HSP genes were identified, and 100 among them were up-regulated (Fig. 6B). Small heat shock family (HSP20) was the major DE group found in this study followed by the HSP70, HSP100 and HSP90 group. We also identified twenty-nine significantly DE unigenes related to the cytochrome (*CYP*) gene family, while 75 were related to photosynthesis and light reaction function as revealed by fold change analysis. All of them were down-regulated under drought stress including *CAB1* (chlorophyll A/B binding protein 1 and 6), *LHB1B1* light-harvesting chlorophyll-protein complex II subunit B1, *PSAD* -2, *PSAF*, *PSAG*, *PSAH2*, *PSAK*, *PSAL*, *PSAN* (involved in photosystem I), *PSB* group with subunits (components of photosystem II) and others related to *ATPase* synthesis (Supplementary Dataset 4-S2).

### SSR and SNP detection

The high-throughput transcriptome sequencing provides excellent resources toward the discovery of cost-effective and polymorphic genetic markers (SSRs, SNPs, Indel). We identified total 13,375 SSR markers by using MISA tool within 12,279 unigenes in *A. sisalana* transcriptome (Supplementary Dataset 5-S1). The average density of microsatellites was found to be one SSR per2.9 kb. Based on the motif repetition, these microsatellites were further categorized into mononucleotide (5318), followed by di-(4347), tri-(3544), tetra-(97), Penta-(37) and hexanucleotides motifs (32), while about 1096 were present in the compound formation (Table 2). (A/T)n motif was the dominant for mononucleotide, (GA/CT/AG)_n_ for di- while (TGC/GAG)_n_ for trinucleotide microsatellites. Specific primers were designed for these SSRs by using Primer3 software and 8164 SSR was verified for a single amplification by in silico PCR with 100-280bp product size (Supplementary Dataset 5-S2). SNPs endure the ability to produces high-density genetic maps, association mapping and molecular markers with the promise of lower cost and error rate. In this study, putative variants were called by aligning the raw reads with the non-redundant *de novo* assembled reference database. In total 36,525 high confidence variants position were identified includes 35,059 and 1466 for SNPs and indels respectively in 17363 unigenes (Supplementary Dataset 6-S1). An average frequency between all the SNPs in these unigenes was 330 bp. The paleopolyploid nature of *A. sisalana* may increase the possibility of high counts of SNPs due to the identical paralogous loci in the genome. Large proportion of the unigenes (9576) had the single base shift than di- (3470), tri- (1901) and tetra- (1051), that accounted 27.3%, 9.8%, 5.4% and 2.9% respectively (Supplementary Dataset 6-S2). Transitions and transversion frequencies including six variations are listed in Table 3. The transition between A and G happen most frequently than any other variation. Validation of these SNPs will be required but their annotation indicates potential polymorphism in drought-regulated transcripts.

**Table 2:** Statistics of SSRs identified in *Agave sisalana*.

SSR mining	
Total number of sequences examined	67,328
Total size of examined sequences (bp)	39203184
Total number of identified SSRs	13375
Number of SSR containing sequences	10729
Number of sequences containing more than one SSR	2108
Number of SSRs present in compound formation	1096
**Distribution of SSRs in different repeat types**	
Mono-nucleotide	5318 (39.7%)
Di-nucleotide	4347 (32.5%)
Tri-nucleotide	3544 (26.4%)
Tetra-nucleotide	97 (0.72%)
Penta-nucleotide	37 (0.28%)
Hexa-nucleotide	32 (0.24%)

**Table 3:** Statistics of identified SNPs.

Number of SNP	
Transition	
A<−>G	10143 (28.9%)
C<−>T	9962 (28.4%)
Total	20105 (57.3%)
Transversion	
T<−>G	3817 (10.88%)
C<−>G	3236 (9.23%)
A<−>T	4239 (12.09%)
A<−>C	3662 (10.4%)
Total	14954 (42.6%)
Total	35059 (100%)

### Validation of the RNA-Seq data

**Figure 7:**
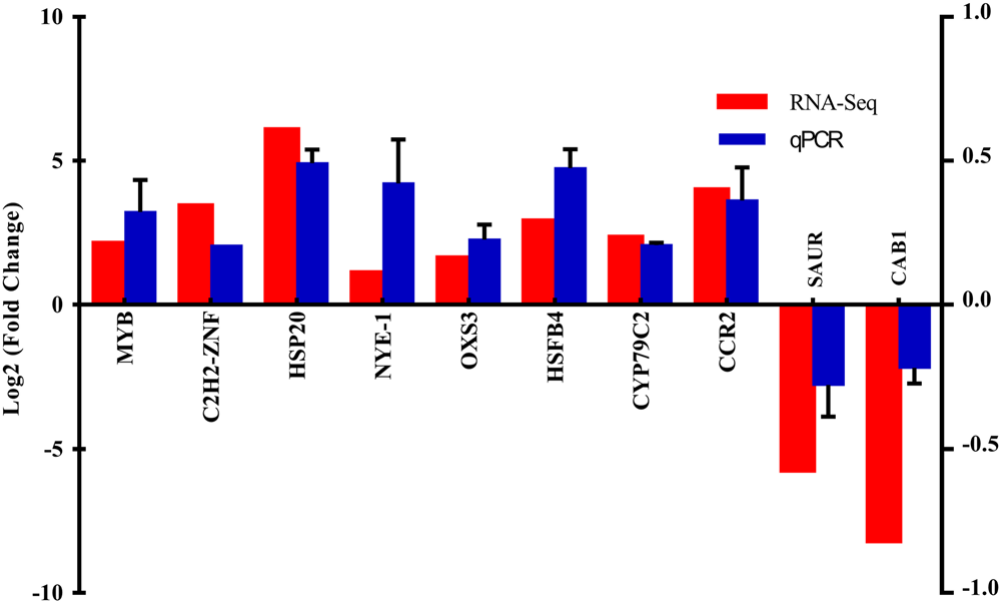
RNA-Seq differentially expressed genes data validation by quantitative real-time PCR (qRT-PCR). (Supplementary File 6 for additional information).

To confirm the reliability of expression data, 20 DEGs were studied by using the quantitative real-time PCR (qRT-PCR). The results showed almost same level of fold changes between RNA-Seq expression and qRT-PCR analyses (Fig. 7).

## Discussion

### Insight into the *de novo* transcriptome assembly and sequence annotation

Drought tolerance is a multi-pronged mechanism orchestrated by a complex set of gene actions in plants. Its understanding requires a comprehensive approach to explore gene expression and physiological and biochemical pathways. To investigate the dynamic variation of *A. sisalana* transcriptome to drought conditions, we employed RNA-seq approach using the Illumina platform. We applied ninety days of drought stress which is considered of sufficient length to activate the plant transcriptome under stress as reported in various studies^12,31^. We observed 18.5% fewer numbers of reads from drought-stressed RNA libraries compared to control data, giving rise to the hypothesis that the *A. sisalana* genome compromised normal growth processes during the drought with significant up-regulation of signaling and regulatory proteins. In total, 90.3% clean reads were assembled into 67,328 non-redundant unigenes that permitted gene annotation and association of transcripts with biological functions. In addition to functional assignment, the high similarity of unigenes to other plant protein sequences also confirms their integrity^32^. With BLAST search, 37,546 unigenes out of 67,328 could be functionally assigned with sequences in the nr database, confirming the reliability of the assembly. Insufficient information in public databases and high sequence variations in agave species could arguably be the reasons underlying the high number of un-assigned sequence in *A. sisalana* genome. GO-based providing information on biological roles^33^ of transcripts under drought stress was deciding to identify the drought tolerance mechanisms, including the discovery of novel drought stress-related genes in *A. sisalana*^33^. Based on differential expression analysis, a number of genes and pathways interactions have been categorized into functional and regulatory groups.

### Induction of Functional proteins in drought response

#### Heat Shock Proteins (HSPs)

The plant’s capability to resist environmental strains is central to development. Protein dysfunctioning is a routine event under the abiotic stress and it is extremely crucial to keep protein functional under the stress. The trigger of the heat shock proteins is the most prominent response to depreciate the cellular injuries and reestablishment of cellular homeostasis. Categorization of these proteins is based on their approximate molecular weight (Hsp100, Hsp90, Hsp70, Hsp60, and HSP20, the small Hsp (sHsp) families)^34^. In this study, fifty-three heat shock proteins unigenes from six major families includes the sHSP20 group members (HSP17.4, HSP17.6II, HSP18.2, HSP21, ATHSP22.0, and HSP23.6-MITO), HSP70, HSP90.1 and HSP10I were up-regulated under drought stress. HSP-20 was the significantly enriched group with the 8.2 enrichment score including the homolog of *Arabidopsis* (ATHSP22.0, AT5G51440, AT2G29500, AT1G52560) (Supplementary Dataset 4 S5). sHSPs are the ubiquitous proteins and can be triggered by multiple stresses includes water stress, high temperature, heavy metals, and toxic substances. More than 300-fold expression of small heat shock proteins was observed in *S. oleracea* under heat stress^25^. In *Arabidopsis*, overexpression of GmHsp90s family from *Glycine max* act as a damage control agent under the abiotic stress^35^.

### Antioxidants response and Osmotic adjustments to dehydration

Over-production of ROS is extremely harmful to plants as it causes lipid peroxidation, DNA damage, and programmed cell death^36^. The antioxidant enzymes constitute the “first line of defense” against these damages. Here in the study, induced expression of enzymatic and non-enzymatic scavenging molecules indicates the active protection shield against the oxidative stress in *A. sisalan* leaves. The enriched GO categories like “response to abiotic stimulus” (GO:0009628) and “response to stress” (GO:0009628) give us a strong clue about the active antioxidant enzyme mechanism. Sixteen unigenes were associated in enzymatic scavenging include catalase (*CAT1, CAT2*), ascorbate peroxidase (*APX2, APX4, TL29*), peroxidase (*PER64, PAP10*) and glutathione (Supplementary Dataset 4 S6). Two unigenes (DN17768_c0_g1_i2 and DN19391_c0_g2_i2) encodes the ascorbate peroxidase 2 (*APX2*) and ascorbate peroxidase 4 (*TL29*) groups, while others four were homolog to *AT1G71695 (*Peroxidase superfamily protein), *AT2G41480 (PRX25*)*, AT5G66390 (PRX72*)*, and AT4G33420* (*PRX47*). Ascorbate peroxidase has a significant role in the ascorbate-glutathione detoxification system. GST (ec:2.5.1.18) is critical in glutathione metabolism and is considered as an important indicator for improving the tolerance capability of rice and *Arabidopsis*^37^. Genes that encode enzymes (ec:1.8.5.1), glutathione dehydrogenase (ascorbate) and (ec:2.5.1.18) glutathione S-transferase taking part in glutathione metabolism were also detected. Heavy metals accumulation in plants is highly reactive and lethal to living cells. The detoxification transporters and their proteins are well known to detoxify the heavy metals into vacuole of cells and maintain them in a balanced amount^38^. Total sixteen genes were identified, 6 were associated with the pleiotropic drug resistance-type ATP-binding protein- PDR (PDR4, *PDR5*, *PDR12*), two tonoplasts based heavy metal ATPase 2 (HMA2), one was related to farnesylated protein 6 (FP6), others included heavy metal-associated isoprenylated plant protein (*HIPP22, HIPP27*). In *A. thaliana*, members of HIPP family involved in cadmium transport play a role in cadmium detoxification^36,39^.

Osmolytes are the nontoxic small compounds that are synthesized and accumulated in plants under abiotic stress. These include non-toxic macromolecules; organic compounds, sugars, sugar alcohols, starch, lipid peroxidase and free proline^40^. We also observed significant activities in the metabolism of sugar and starch (non-sugar) related enzymes. The involved pathway was also enriched, “ko00500” with involvement of seven upregulated transcripts. The associated enzymes within this pathway were (ec:3.2.1.21) beta-glucosidase/gentiobiase, (ec:3.2.1.2) saccharogen amylase/beta-amylase, (ec:3.1.3.12) trehalose-6-phosphatase/trehalose-6-phosphate phosphohydrolase and (ec:3.2.1.48) sucrose sucrase/alpha-glucosidase. Trehalose 6-phosphatase (TPP/TPS) is a key player in osmoregulation which strengthens the tolerance in plants to the drought stress^25,41^. We noted up-regulated six genes “*TPS2, TPS3*, and *TPS6”* encoding the trehalose enzymes. Enzyme phosphorylase (ec: 2.4.1.1) was also altered that take part in the decomposition of non-sugar molecules under drought stress. Other enzymes like “(ec:3.1.1.11) - pectinesterase/pectin-demethylase” and “(ec:3.2.1.15)-pectinase/pectin depolymerase were induced under drought stress in this study. They are involved to enhance the cell-to-cell adhesion, cell elongation, the porosity of the wall, disease resistance and ultimately plant growth and development. The role of secondary metabolites like flavonoids, phenylpropanoids are also critical under osmotic stress. Phenylpropanoid biosynthesis pathway (ko00940) was enriched with upregulated enzymes, (ec:2.1.1.146)-O-methyltransferase and (ec:1.11.1.7) peroxidase/lactoperoxidase. Induction of these enzymes indicate the critical role in phenylpropanoid biosynthesis in the osmotic stress. No significant change in expression related to proline biosynthesis was  observed.

### Cuticle, wax biosynthesis, cell wall metabolism under drought stress

Wax accumulation on outer surface of plant cuticle provides the hydrophobic protection against water loss under osmotic stress^42^. Biosynthesis of wax begins in epidermal cells of plastids with a C16-C18 long chain of fatty acid with cofactor acyl carrier protein. β-ketoacyl-CoA synthase (*KCS*), β-ketoacyl-CoA reductase (*KCR*), β-hydroxy acyl-CoA dehydratase (*HCD*), and enoyl-CoA reductase (ECR) catalyzed the long chain to produce very long chain fatty acid (*VLCFAs*). In this investigation, all core-mentioned enzymes that take part in wax biosynthesis and regulation were found in the differentially expressed database except the *HCD* (Supplementary Dataset 4-S12). The expression of *KCS6*, *GPAT1* (Glycerol-3-phosphate acyltransferase 1) and LTP3 (Lipid transfer proteins 3) were induced under drought stress along with *CER1* (Eceriferum /trans-2-enoyl-CoA reductase 1) and *EXL 2* and 3 (EXORDIUM like 2). (ec:2.3.1.75) long-chain- alcohol O-fatty-acyltransferase; and (ec:2.3.1.20) palmitoyl-CoA-sn-1,2-diacylglycerol acyltransferase. These enzymes take part in cutin, suberine, and wax biosynthesis pathway (Ko00073) with (ec:1.14.13.8 – monooxygenase) were also upregulated under drought stress. Surprisingly a long list of wax biosynthesis genes was also down-regulated under drought stress (Supplementary Dataset 4-S12). Muthusamy *et al*. and Ni *et al*. also reported a high proportion of downregulation of wax biosynthesis transcripts under drought stress^43,44^.

The ABC transporter G subfamily has been reported to be involved in the export of mature fatty acids in *A. thaliana*^45,46^ (Supplementary Dataset 4-S8). The involvement of *ERF*/*AP2* has been extensively reported in the cuticle biosynthesis, especially regulation, accumulation, and transport in response to the abiotic stresses^23^. *MYB* TFs are also characterized for their role in the cuticle metabolism^44^. These factors in combination with other regulatory genes in *A. sisalana* may act as the coordinator for leaf cuticle synthesis. Identification of these wax related genes would assist further to understand the biosynthesis and functions of the cuticular wax under drought stress.

### Signaling and Regulatory Proteins Response to Drought Stress

#### Ca^+^ Signaling and activation of kinases (PK & RLK)

Activation of various signaling transduction pathways is key phenomena that happen mostly across the cell membranes to initiate a series of self-protective mechanisms under unfavorable conditions within the cells. Proteins and receptor kinases are the sensors on the membrane of the cell that perceive extracellular signals and transmit them to target genes for the activation of specific stress response. Abundance of the kinases is expected as their domain is actively involved in a number of cellular processes. In this study, seventy-eight significantly differentially expressed transcripts were associated with the protein and receptor kinase group under drought stress conditions. Majority of them belong to PK and RLK superfamily, like Leucine-rich repeat protein kinase and Leucine-rich receptor-like protein kinase family protein RLKs (*BAM 1 & 2, BRII, CLVI, ER*, and *FSL2*). The other includes adenosine kinase (*ADK 1 & 2*, *CBL* and *CBL)* interacting protein kinases (*CIPK1 & 3, CRCK2*), SNF1-related (*SNRK2* 0.1), Serine/Threonine kinase catalytic domain protein (*NEK5*) and other (Supplementary Dataset 4-S4). Leucine-rich receptor-like kinases (RLKs) are one of the major group that managed the meristem proliferation, reproduction, organ initiation, specification and hormonal signal cascade. There are several reports that revealed their response towards the drought tolerance e.g. in *Arabidopsis* abrupt increased of RLKs was observed towards the osmotic stress^47^.

The abrupt increase in the calcium ions happens in plants under abiotic stress conditions, is a sign of activation of the stress-responsive cascades. In this study, nine significantly enriched unigenes that belong to the calcium transport signaling group includes, calcium ion binding protein (*SUB*, *SUB1*), calcium exchanger (*CAX3*, *CAX5*, and *CAX7*) and tonoplast calcium sensor (*CBL3*) have been identified (Supplementary Dataset 4-S3). The induced response of these proteins under drought stress stabilized the structural rigidity of cell wall. The *CAX* group of genes has been discovered in a number of plant species and act ubiquitously as they regulate the tonoplast localized Ca 2+/H + antiport activities. Furthermore, the interaction among different protein phosphatases like *HAI2*, *HAI3* and kinases such as serine/threonine-protein kinase (NEK5), CBL-interacting protein kinase initiated the protein phosphorylation cascade which take part in cell signal recognition and transduction in the responses to abiotic stress^48^. SNF1-related protein kinase 2.1 (SNRK2.1) also act as a positive regulator of the hormonal (ABA) signaling. In *A. thaliana* complex association between the Calcineurin like proteins (CBL4/CIPK) are associated with the sodium ions release from the cells and absorption of K^+^ by the root surface, that regulates the stomatal behavior under osmotic stress^49^.

#### Phytohormones pathways gene to drought stress

To combat various environmental stresses, novel and dynamic approaches should be devised, and phytohormone engineering could be a method of choice to improve the productivity including drought resistance^50^. Plant hormones improve the resistance to osmotic stress by regulating the physiological process. The abscisic acid (ABA) is a key plant growth regulator that directly involved in the responses to abiotic stresses^50^. Here, we noted twenty-three up-regulated unigenes related to *ABA*-induced protein phosphatase 2 and 3 (*HAI2* and *HAI3*) (ec:3.1.3.16), one protein phosphatase 2CA group (*PP2CA*) (ec:3.1.3.16), homolog of *ABI2* (*HAB, HAB2*), while four with protein phosphatase 2C families (*ABI1)* that were homologous to *AT3G62260*, *AT3G63320*, *AT1G18030*, *AT3G12620* IDs. A higher number of up regulations of the ABA encodes unigenes including ABA receptor family (PYL4) is an indication of accumulation of ABA due to the decreased cellular water contents under drought stress (Supplementary Dataset 4-S9). Protein phosphatases are the chief regulators and are considered to mediate the ABA triggered signaling pathways. Induced *PP2C* and *PP3C (*Protein phosphatases) level in association with the ABA pathway indicated its hyper response to the drought stress in *A. sisalana*, which is a conserved mechanism in the metabolism of ABA. The differential expression of these genes may regulate the guard’s cell of stomata for gaseous exchange and activation of ABA-dependent regulatory elements, such as MYB factors.

Auxin biosynthesis and transport are essential in regulating the response to environmental stresses, including drought, salinity, and pathogen attack. Changes in Indole-3-acetic acid (IAA) biosynthesis in response to external stimulus regulate the stomatal closure via cross-talk with other plant hormones like ABA and others. The IAA mutant plant of Arabidopsis exhibited significant induced water loss than the normal plants^51^. In this study, gene enrichment analysis showed the number of genes contributing to the growth under drought stress related to auxin hormones including auxin-induced protein (*IAA13, IAA16, IAA33*), auxin response factor (ARF-1,9,11,19) that were homologous to AT1G19220, *AT4G23980*, *AT1G59750, AT2G46530*, GH3 and 4, auxin efflux carrier family protein (PIN1and EIR1), like-LAX2 related gene, auxin- responsive factor AUX/IAA-like protein (NPH4) and auxin binding ABP like proteins (Supplementary Dataset 4-S10). Several positively regulated induced auxins genes is an indication of there important role in *A. sisalana* against drought stress.

We also observed thirty-seven DE unigenes related to the cytochrome p450s gene family (Supplementary Dataset 4-S11). Cytochrome is one of the largest and central superfamilies in plants, so far encoding about 1% of the protein coding sequences that act in hormonal control mechanisms including biosynthesis and catabolism of primary and secondary metabolites^52^. Several members of this group like *CYP71* are known to catalyze the production of aliphatic and aromatic nitriles suggesting their possible role in the defense to the biotic stress^53,54^. Members of CYP86, CYP94, CYP96, and CYP704 are also known as candidates for cuticle biosynthesis^55,56^. The detection of these cytochromes in our dataset may indicate their potential role for cuticle biosynthetic . The promoter region of various cytochrome genes has the affinity for the drought-induced TF includes MYB/MYC, TGA, and W-box for the WRKY. The appearance of high number unigenes associated with these TFs and the CYPs-450 might be a strategy to combat stress. Biosynthesis of jasmonic acid (JA) and Brassinosteroids (BR) hormones are also stressed sensitive. In our data, we noted two differentially expressed genes involved in the alpha-Linolenic acid metabolism that regulate the JA biosynthesis^57^. There are several reports that confirmed their involvements to improve the stress tolerance ability of drought-tolerant cultivars^58,59^. The transcription factor-like *MYC2* is a key regulator of JA response and their upregulation in this study indicates its regulatory role in this process and act as a mediator in cross-talk along with WRKY and MYB TFs.

#### Transcriptional regulatory network induced a response to drought stress

TFs are the key regulatory switches that directly regulate the signal transduction pathways^60^. In eukaryotes, especially in plants, TFs are highly conserved and represented by various multigene families to perform specific functions. The number of genes encoding these families may vary due to origin, expansion, and tissue-specific functions. In the current study, 372 transcription factors belonging to the ERF (*E2F3*) family, bHLH, NAC, HSF, MYB and Zinc finger-like protein and others were found to be differentially upregulated under drought stress (Supplementary Dataset 6 S1). In addition, we also found two transcription factors of the GRAS family, *PAT1*, and SCL7 (homologs of *AT5G41920). GRAS plays* a critical function in plant growth and environmental adaptations, especially in the modulation of plant tolerance to stress^61^. In *A.thaliana*, up-regulation of the SCL7 and SCL 23 TFs has enhanced tolerance to the salt and drought stress^58^. Heat Shock TFs (HSF) are central facilitator for expression of the genes responsive to various abiotic stress conditions. Here, eight induced DEG got annotation to HSFs group, including *HSF3*, *HSFA3*, *HSF*-*A4A*, *HSFB2A*, *HSFB4*, and *HSFC1*. *NAC* proteins are plant-specific TFs that are considered important for plant development, abiotic stress responses as well as for ABA signaling. The Arabidopsis and rice genome hold 106 and 149 NAC proteins respectively. Here we found 27 induced *AsNAC* related TFs to drought stress. Overexpression of NAC proteins enhanced the longevity and abiotic stress tolerance efficiency in *Arabidopsis*, *Oryza sativa, Zea mays* (*ZmNAC55)* and cicer (*CarNAC4)*^62^.

## Conclusion

To the best of our knowledge, here we reported the first transcriptome study of *Agave sisalana* with the objective to identify the functional genes associated with drought tolerance. 67328 unigenes were *de novo* assembled, and 37546 were functionally annotated. Further differential gene expression provides the clear understanding of responsive mechanism to drought stress. In addition, the identified genetic marker will provide the source for marker development in this species. This study may not only provide the insights to genomics of adaptation of drought tolerance in agave but also excellent genetic resources for drought tolerance crop development.

## Materials and Methods

### Plant material, stress conditions, and tissue sampling

The offshoots of similar age/height from 1-year-old mature adult *A. sisalana* plants, which were asexually propagated from a single “the mother plant,” were used for the current study (Supplementary Information 3-S1). These offshoots were further grown in pots (one per pot) having the soil mixture of peat moss, vermiculite and sandy soil in the ratio (1:1:1) in the greenhouse. After 90 days of propagation, we divided these plants into two groups randomly with three replicates each. A control group (C); watered regularly while the other treated group (T); no water was applied until the leaf sampling. The newly emerged middle leaves of the *A. sisalana* rosette (Supplementary Information 3-S2) were harvested from each plant of the group, immersed into liquid nitrogen, ground well to a very fine powder and stored at −80 °C till further molecular investigation.

### RNA isolation, cDNA library preparation, and Illumina sequencing

Total RNA was isolated from the stored ground leaves by using the Trizol method with column based purification as described by^34^. Genomic DNA contamination was removed by RNase free amplification grade DNase I kit (AMPD1-sigma). TruSeq RNA Sample Prep Kit v2 (Illumina, Inc. San Diego, CA, USA) protocol was used for library constructions. Six paired-end cDNA libraries were constructed and sequenced on the Illumina HiSeq^TM^2500 platform with the 101 bp insert size at Macrogen Inc. (Korea). The FastQC (http://www.bioinformatics.babraham.ac.uk/projects/fastqc/) application and NGS QC Toolkit (http://www.nipgr.res.in/ngsqctoolkit.html) was used to ensure the standard quality statistic for the FASTQ files.

### Transcriptome *de novo* assembly and evaluation

The high quality, adapter free reads were used to construct the *de novo* assembly with assemblers including Trinity v 2.3.1 (https://github.com/trinityrnaseq/trinityrnaseq/wiki) under the default settings (25 K-mer), Trans-ABySS (http://www.bcgsc.ca/platform/bioinfo/software/trans-abyss) with multi K-mer adjustment to include odd numbers, i.e., 23 k-mer, 25, 27 and so on up to 63 k-mer, and Short Oligonucleotide Analysis Package (SOAP) (http://soap.genomics.org.cn/SOAPdenovo-Trans.html). Quality evaluation of assemblies was considered with major bioinformatics indicators like contigs mean length, GC percentage, N50, and N25 value. We also compared the mean distribution plot of the contigs produced by aforementioned assemblers with the *Ananas comosus* V3 partial genome assembly obtained from (https://phytozome.jgi.doe.gov/pz/portal.html#!info?alias=Org_Acomosus_er) and *A. deserti* transcriptome data (http://datadryad.org/resource/doi:10.5061/dryad.h5t68). Based on these indicators, the Trinity developed assembly was selected for further downstream investigations. Further unigene decontamination was done using MEGAN version 6.01 (http://ab.inf.uni-tuebingen.de/software/megan/) on taxonomy ID basis. The longest isoform (unigenes) was generated by combining the contigs having consensus sequences by Perl script obtained from google group “Trinity RNAseq-user” by Brian Haas (https://groups.google.com/forum/#!forum/trinityrnaseq-users). Assembly quality was assessed using three approaches. (i) a Perl script ORFpredictor (ii) Reads mapping back to transcriptome (RMBT) (iii) BUSCO v1.161 (**B**enchmarking **U**niversal **S**ingle-**C**opy **O**rthologs) analysis.

### Transcript annotation and functional classification

Cleaned contigs were annotated using the NCBI standalone local BLASTX Programme with the cutoff e-value 10^−5^ against the NCBI nr database (ftp://ftp.ncbi.nlm.nih.gov/blast/db/FASTA /), SwissProt/uniport (http://web.expasy.org/docs/swiss-prot_guideline.html), Viridiplantae database taxonomy ID (tax ID: 33090) (http://www.uniprot.org/taxonomy/33090), COG database https://www.ncbi.nlm.nih.gov/COG/ and plant TFdatabase (http://planttfdb.cbi.pku.edu.cn/) by homology search. The Kyoto Encyclopedia of Genes and Genomes (http://www.genome.jp/kegg/) was accessed for biochemical pathway identification based on the assigned enzyme codes. The best BLAST hits were used to choose the downstream analysis direction. GO analysis was performed in the standalone Blast2GO v3.2 (https://www.blast2go.com) with value 1e^−3^, annotation cutoff filter 55, code set to 0.8 to assign the GO terms to each transcript regarding molecular functions (MFs), biological processes (BPs), and cellular components (CCs). GO enrichment analysis was carried out by AgriGO software with FDR value not less than <0.05.

### Transcript count and differentially expressed gene identification

First, the abundance of each transcript was calculated by bowtie2 and RSEM (RNA-Seq by Expectation-Maximization-http://deweylab.github.io/RSEM/package) for each library. Differentially expressed genes (DEGs) among the drought and control treated libraries were calculated by using the Empirical Analysis of Digital Gene Expression (edgeR) (http://bioconductor.org/packages/release/bioc/html/edgeR.html) statistical package. The trimmed mean of M-values (TMM) method was used to calculate the normalization factors. The threshold p-value < 0.05 and false discovery rate (FDR) < 0.001 was adjusted to identify the differentially expressed genes by fold change ( ≥ 1).

### Simple Sequence Repeat (SSR) and Single-Nucleotide Polymorphism (SSR) Calling

Perl supported script MISA (MIcroSAtellite identification tool- http://pgrc.ipk-gatersleben.de/misa/) was used to mining the SSR repeats with di-, tri-, tetra-, penta- and hexanucleotide motifs present in the *A. sisalana* assembly. The latest version of PRIMER3 with modifies Perl scripts (p3_in_v2.pl p3_out_v2.pl https://gist.github.com/ascatanach/7a562621b9c86c7b5e81973136e6419f) was used for primer designing. Clean reads from the six libraries were aligned back to the unigenes by short read aligner (bowtie2) with default parameter^63^. Further SNPs and indel calling was carried out using the mpileup function of SAMtools (http://samtools.sourceforge.net/) and VarScan (http://varscan.sourceforge.net/) mpileup v0.1.7a^64,65^.

### Quantitative RT-qPCR validation

We randomly selected 10 annotated DEGs to verify the RNA-Seq expression data. Gene-specific primers were designed from the selected unigene sequences using Primer 6.0 software (http://www.premierbiosoft.com/primerdesign/) (Supplementary Information 4). Relative fold expression (RT-qPCR) was carried out on the IQ5 system (BioRad) by using the SYBR® Green PCR Master Mix (cat#4309155). Thermal settings included the following conditions: 95 °C for 3 min, followed by 40 cycles at 95 °C for 30 s, then 60 °C for 30 s and at 72 °C for 30 s. All this study was carried out on three independent biological and technical replicates. Relative Expression Software Tool (REST) (http://www.gene-quantification.com/rest-2009.html) was used for relative fold expression calculation.

## Electronic supplementary material

Supplementary information 1

Supplementary information 2

supplementary information 3

supplementary information 4

Supplementary Dataset 1

Supplementary Dataset 2

Supplementary Dataset 3

Supplementary Dataset 4

Supplementary Dataset 5

Supplementary Dataset 6
