# Supplementary material for: De novo assembly of Agave sisalana transcriptome in response to drought stress provides insight into the tolerance mechanisms
Source: Sci Rep. 2019 Jan 23;9:396. doi: 10.1038/s41598-018-35891-6 (PMC6344536; doi:10.1038/s41598-018-35891-6)

Supplementary information 1a and 1b: (a) Illumina platform generated sequencing reads statistics. (b) Experimental Design for RNA-Seq data analysis under control and drought stress condition.

De novo assembly of Agave sisalana transcriptome in response to drought stress provides insight into the tolerance mechanisms

[1,2]Muhammad Bilal Sarwar, Zarnab Ahmad[1], [*] Bushra Rashid[1], [1]Sameera Hassan, [2]Per L. Gregersen, [2]Maria De la O Leyva, [2]Istvan Nagy, [2]Torben Asp, [1]Tayyab Husnain

[1]Plant Genomics Lab, Center of Excellence in Molecular Biology, University of the Punjab, 87-West Canal Bank Road Thokar Niaz Baig, Lahore-53700, Pakistan.

[2] Department of Molecular Biology and Genetics, Aarhus University, Forsøgsvej 1, Slagelse Denmark.

[*]**Corresponding author:** Bushra Rashid

Tel.: +92 (42) 35293141-46; **Fax**: +92 (42) 35293149
E-mail: bushra.cemb@pu.edu.pk

Sr No.	File	Name	size (GB)	total_length_of_read	Total reads	Total read bases (bp)	GC (%)	AT(%)	Q20(%)	Q30(%)
1	C1	1.fastq	5.71	101	43,520,248	4,395,545,048	48.6	51.4	97.899	96.406
2		2.fastq	5.71	101						
3	C2	1.fastq	7.07	101	53,866,820	5,440,548,820	48.2	51.87	97.822	96.282
4		2.fastq	7.07	101						
5	C3	1.fastq	7.21	101	55,165,992	5,571,765,192	48.07	51.93	97.745	96.135
6		2.fastq	7.21	101						
7	T1	1.fastq	5.8	101	44,338,502	4,478,188,702	47.5	52.48	97.78	96.389
8		2.fastq	5.8	101						
9	T2	1.fastq	5.3	101	40,564,456	4,097,010,056	48.1	51.9	97.737	96.123
10		2.fastq	5.3	101						
11	T3	1.fastq	5.15	101	39,389,772	3,978,366,972	48.2	51.97	97.299	95.423
12		2.fastq	5.15	101						
	Total		72.49	101	276,845,790	27,961,424,790	48.11	51.93	97.71	96.13

Experimental Desgin and pipeline used in this study for RNA-Seq data analysis under control anddrought stress condition.

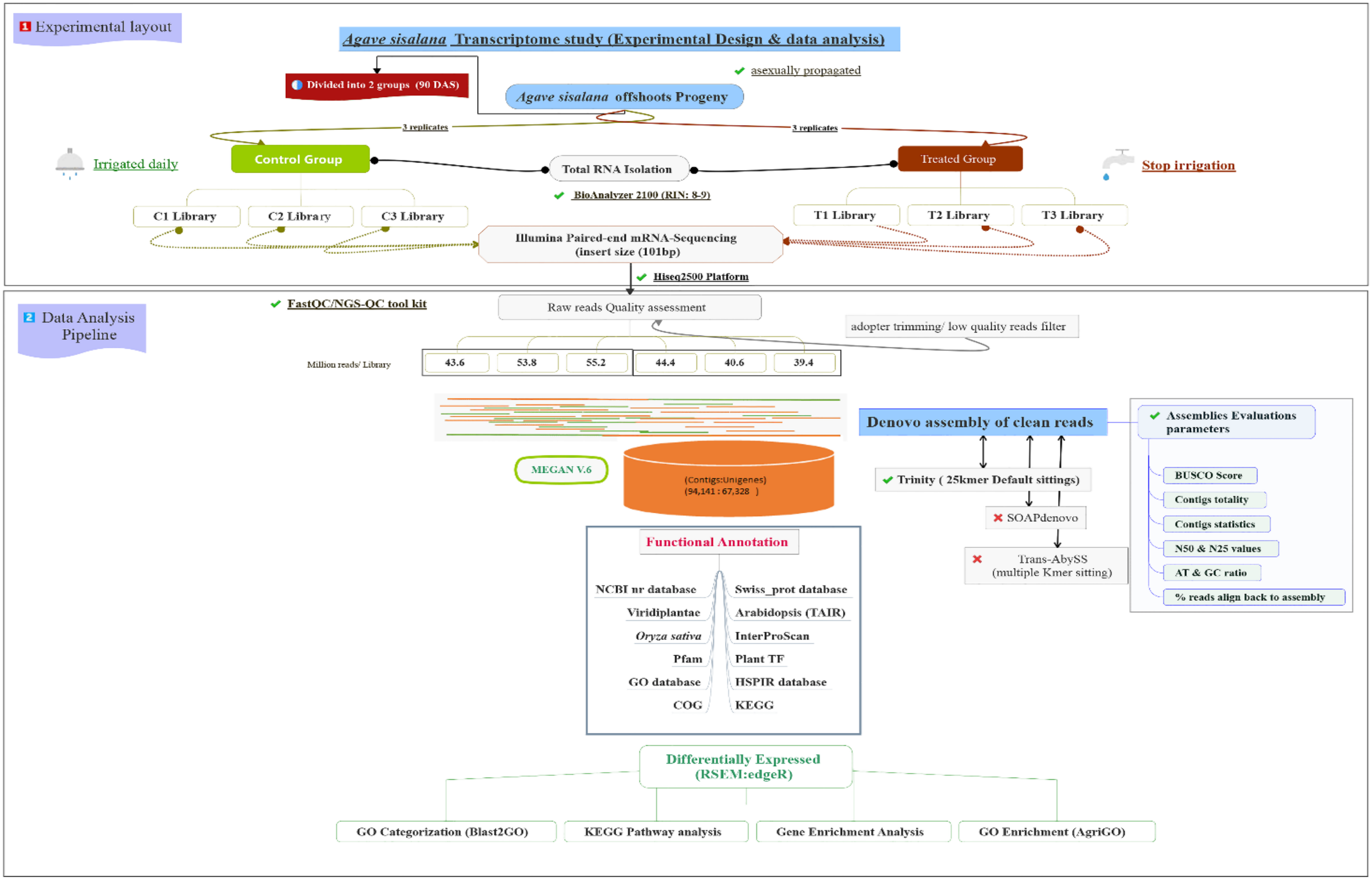

Supplement: Supplementary file 1 — Supplementary information 1 [file 41598_2018_35891_MOESM1_ESM.pdf]
