# Supplementary material for: De novo assembly of Agave sisalana transcriptome in response to drought stress provides insight into the tolerance mechanisms
Source: Sci Rep. 2019 Jan 23;9:396. doi: 10.1038/s41598-018-35891-6 (PMC6344536; doi:10.1038/s41598-018-35891-6)

Supplementary information 2: Statistics related to the differential gene expression including supportive graphs, differentially expressed gene homology against a different database and their functional annotation based on COG database.

De novo assembly of Agave sisalana transcriptome in response to drought stress provides insight into the tolerance mechanisms

[1,2]Muhammad Bilal Sarwar, Zarnab Ahmad[1], [*] Bushra Rashid[1], [1]Sameera Hassan, [2]Per L. Gregersen, [2]Maria De la O Leyva, [2]Istvan Nagy, [2]Torben Asp, [1]Tayyab Husnain

[1]Plant Genomics Lab, Center of Excellence in Molecular Biology, University of the Punjab, 87-West Canal Bank Road Thokar Niaz Baig, Lahore-53700, Pakistan.

[2] Department of Molecular Biology and Genetics, Aarhus University, Forsøgsvej 1, Slagelse Denmark.

[*]**Corresponding author:** Bushra Rashid

Tel.: +92 (42) 35293141-46; **Fax**: +92 (42) 35293149
E-mail: bushra.cemb@pu.edu.pk

S1

[illegible]R	Fold change	Total DEG	Up	Down
0.05	1	8678	3989	4689
0.05	2	2249	761	1488
0.01	1	4616	1842	2774
0.01	2	1861	586	1275
0.001	1	3059	1195	1864
0.001	2	1474	472	1002
0.0001	1	2067	803	1264
0.0001	2	1184	399	785

S2

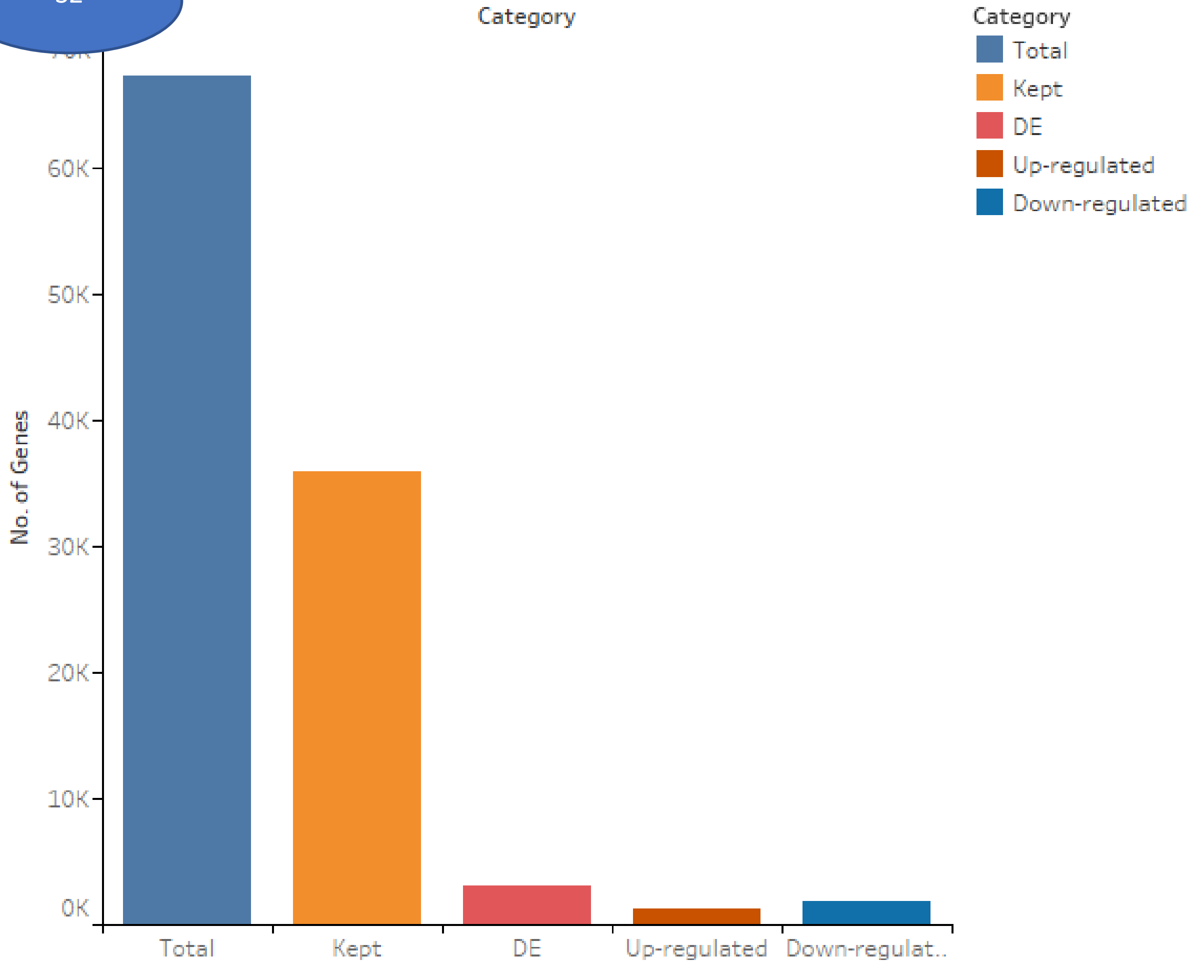

S3

Volcano plot [counts_results]

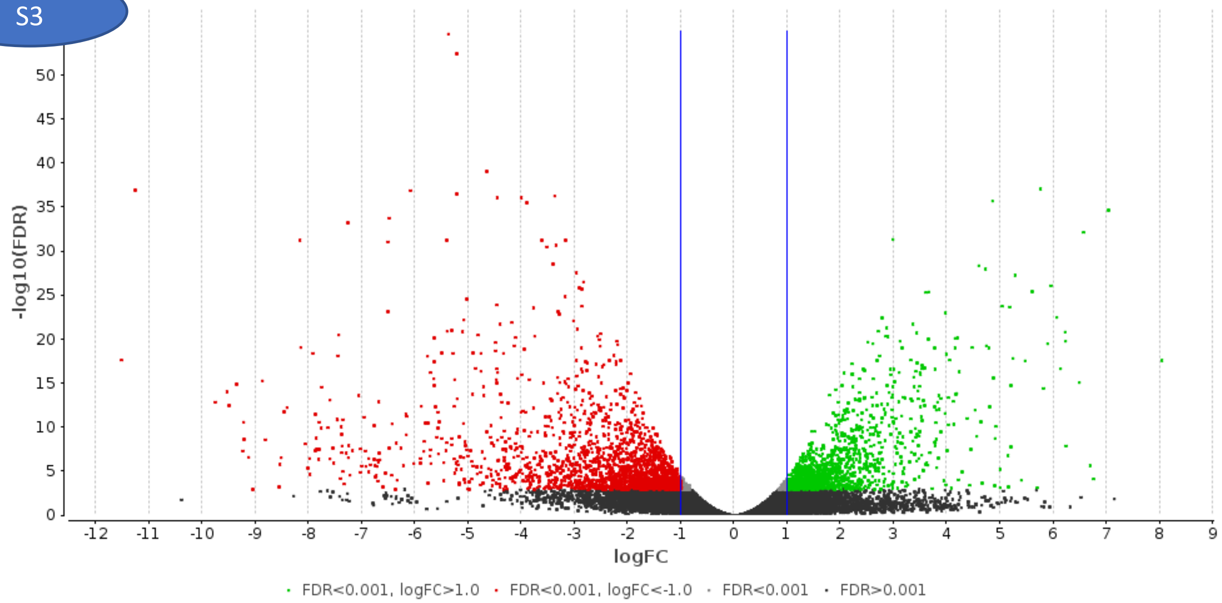

S4

MA plot [counts_results]

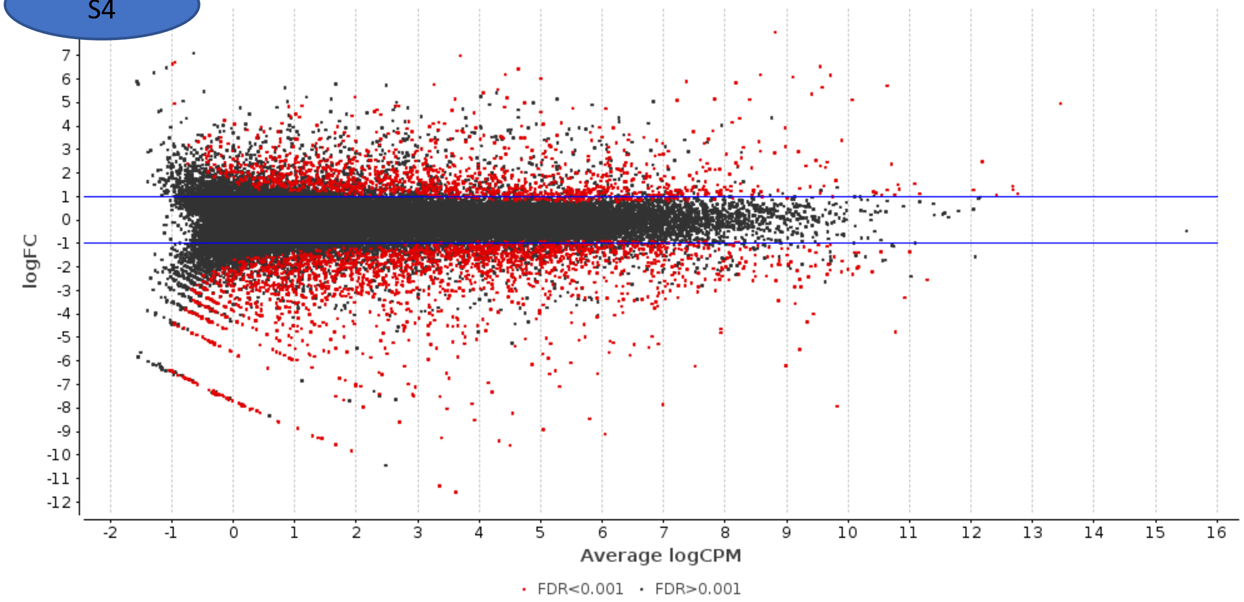

Supplement: Supplementary file 2 — Supplementary information 2 [file 41598_2018_35891_MOESM2_ESM.pdf]
