# Supplementary material for: De novo assembly of Agave sisalana transcriptome in response to drought stress provides insight into the tolerance mechanisms
Source: Sci Rep. 2019 Jan 23;9:396. doi: 10.1038/s41598-018-35891-6 (PMC6344536; doi:10.1038/s41598-018-35891-6)
Supplement: Supplementary file 3 — supplementary information 3 [file 41598_2018_35891_MOESM3_ESM.pdf]

**Supplementary** information 3: Pictorial representation of the offshoot of A. sisalana used for the leaves samplings.

# *De novo assembly of Agave sisalana* transcriptome in response to drought stress provides insight into the tolerance mechanisms

[1,2]*Muhammad Bilal Sarwar, Zarnab Ahmad*[1], [*] *Bushra Rashid*[1], [1]*Sameera Hassan,* [2]*Per L. Gregersen,* [2]*Maria De la O Leyva,* [2]*Istvan Nagy,* [2]*Torben Asp,* [1]*Tayyab Husnain*

[1]Plant Genomics Lab, Center of Excellence in Molecular Biology, University of the Punjab, 87-West Canal Bank Road Thokar Niaz Baig, Lahore-53700, Pakistan.

[2] Department of Molecular Biology and Genetics, Aarhus University, Forsøgsvej 1, Slagelse Denmark.

[*]**Corresponding author:** Bushra Rashid

**Tel.**: +92 (42) 35293141-46; **Fax**: +92 (42) 35293149

E-mail: bushra.cemb@pu.edu.pk

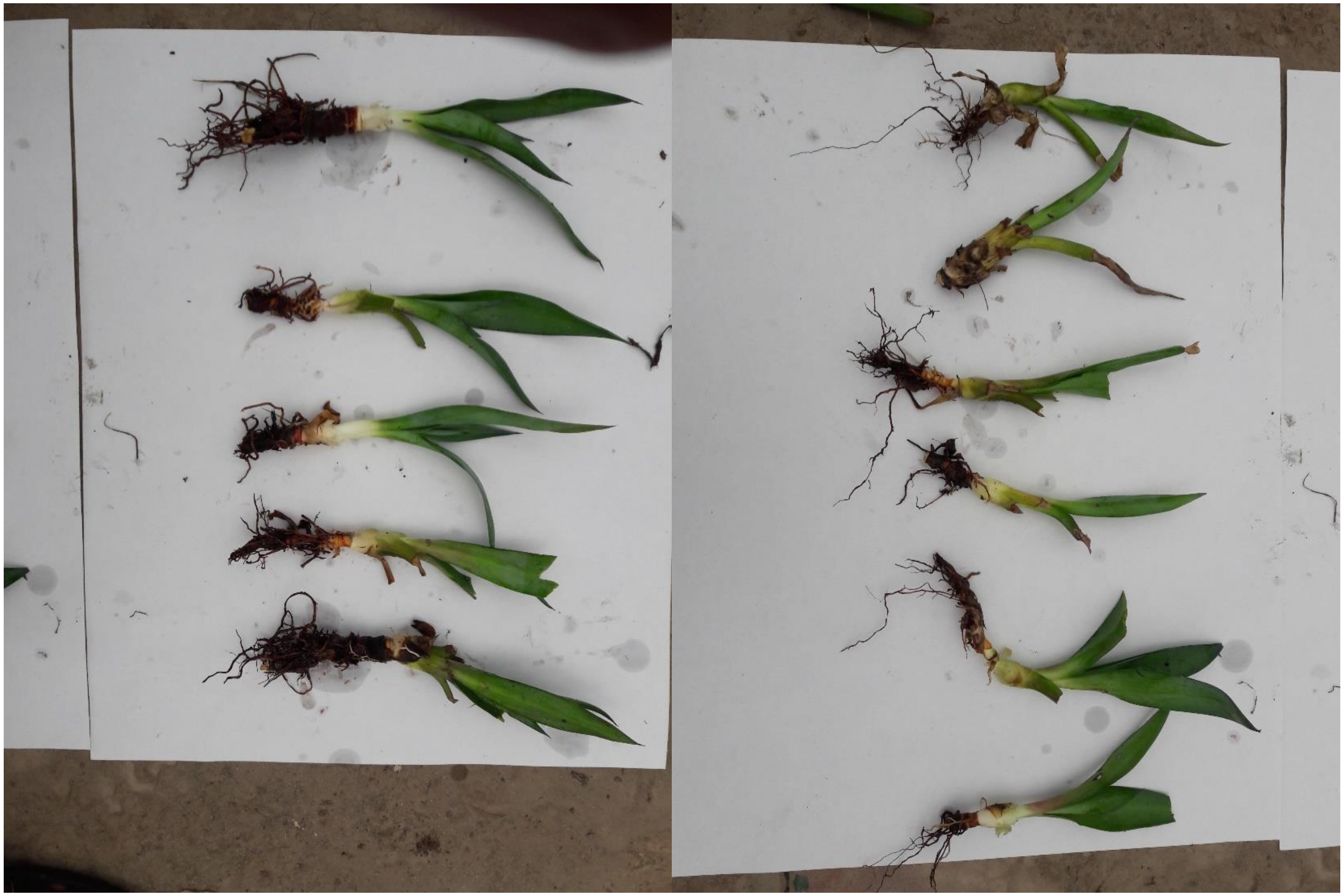

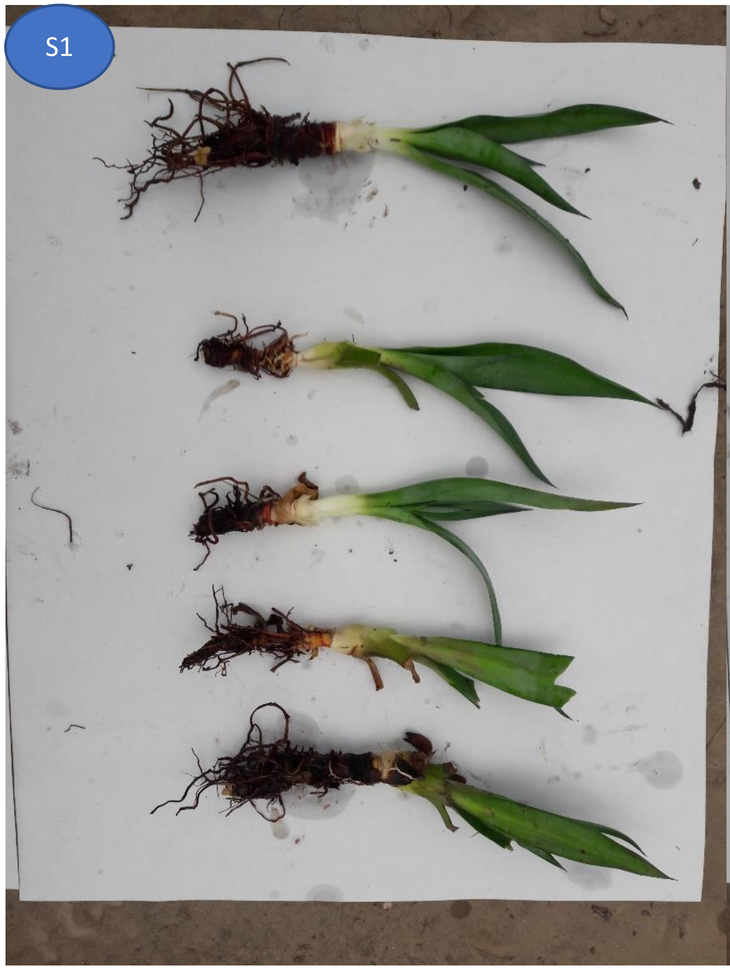
S1

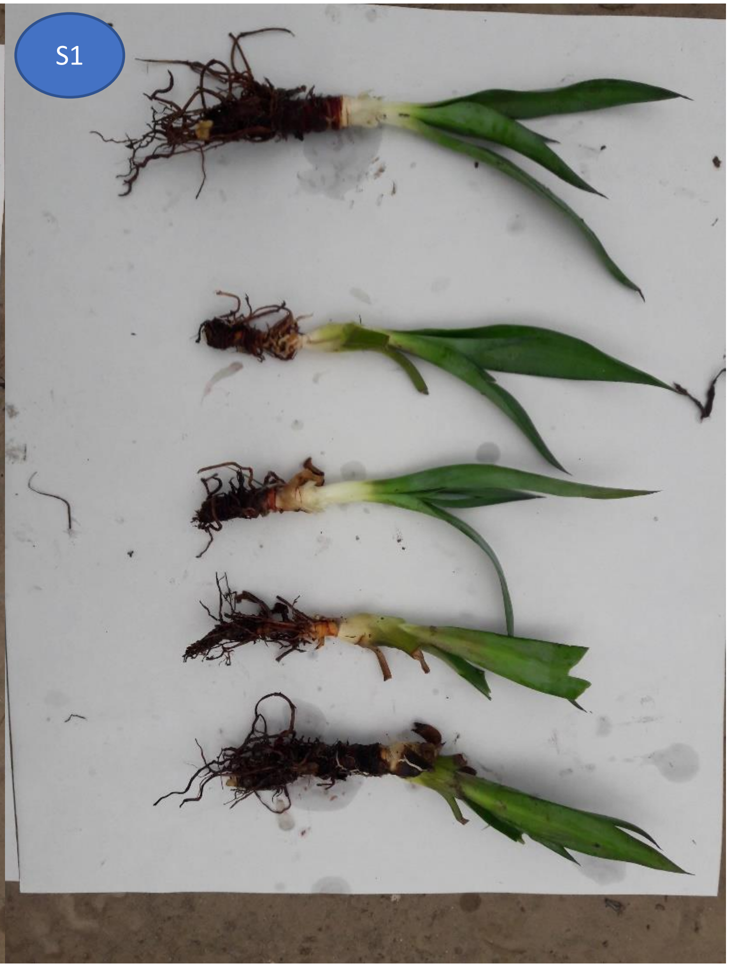
S1

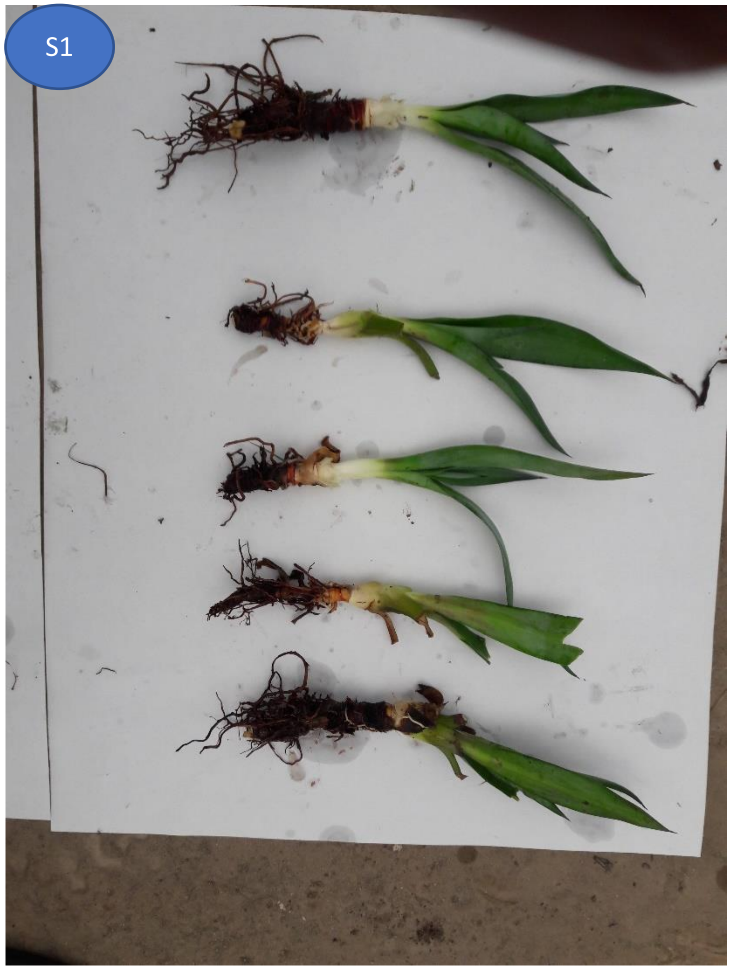
S1

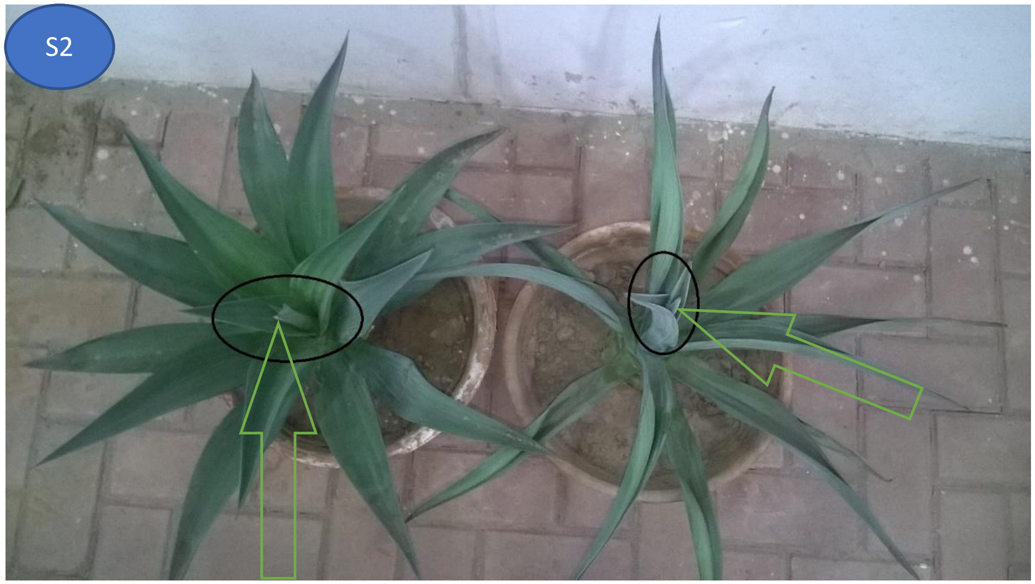
S2
