# Supplementary material for: De novo assembly of Agave sisalana transcriptome in response to drought stress provides insight into the tolerance mechanisms
Source: Sci Rep. 2019 Jan 23;9:396. doi: 10.1038/s41598-018-35891-6 (PMC6344536; doi:10.1038/s41598-018-35891-6)
Supplement: Supplementary file 4 — supplementary information 4 [file 41598_2018_35891_MOESM4_ESM.pdf]

Supplementary information 4: List of the primers used for RNA-Seq data validation by qPCR

De novo assembly of Agave sisalana transcriptome in response to drought stress provides insight into the tolerance mechanisms

[1,2]*Muhammad Bilal Sarwar, Zarnab Ahmad*[1], [*] *Bushra Rashid*[1], [1]*Sameera Hassan,* [2]*Per L. Gregersen,* [2]*Maria De la O Leyva,* [2]*Istvan Nagy,* [2]*Torben Asp,* [1]*Tayyab Husnain*

[1]Plant Genomics Lab, Center of Excellence in Molecular Biology, University of the Punjab, 87-West Canal Bank Road Thokar Niaz Baig, Lahore-53700, Pakistan.

[2] Department of Molecular Biology and Genetics, Aarhus University, Forsøgsvej 1, Slagelse Denmark.

[*]**Corresponding author:** Bushra Rashid

Tel.: +92 (42) 35293141-46; **Fax**: +92 (42) 35293149

E-mail: bushra.cemb@pu.edu.pk

Primers used for quantitative real time PCR analysis.						
**Sequence Definition**	**Sense Primer**	**bp**	**Sequence Definition**	**Anti-sense Primer**	**bp**	**Amplicon Size**
DN43992-F	CTTGAAATACCGAGGCAGTA	20	DN43992-R	AGTGATTGAGGACATGAGTG	20	199
DN16778-F	GATGAAGTCTGGCGTCTC	18	DN16778-R	TCCTCCCTCGTCTTCTTC	18	153
DN30560-F	TCTGAGGAGGAGAAGAGC	18	DN30560-R	AGAGTGTGATGCCAGTTG	18	155
DN624-F	ATGAGCACCTTCAGTTGT	18	DN624-R	CCAGTTGATGGACCTCTC	18	180
DN851-F	CCAGTGCCAGAGATAACG	18	DN851-R	TTCGCCTTCCTTGACATAG	19	148
DN11256-F	TTCTGAGCAGAGCCTTCC	18	DN11256-R	GAGTTGTGCCCGTTGTTG	18	132
DN22781-F	AGAAGGAAGAGGCGTTCA	18	DN22781-R	GGCAGAGTTGTAGAGATGG	19	145
DN10658-F	GCTTCAGTTAAGTGAGTCCT	20	DN10658-R	CGCTAGAGAGGCATACATAG	20	160
DN15873-F	CAGCCATCAGCAAGAACT	18	DN15873-R	GAACCGTTAGAAGAGGAGAT	20	169
DN106-F	GCCTTCGACATCATCACTA	19	DN106-R	CTCTCCGCAGTCAACTATG	19	186
DN32793-F	TCTGAGGACTGCTGGAAT	18	DN32793-R	GTCTTCTGAGCCTGATGTT	19	181
DN17539-F	CGCAATCAAGGCTCATCT	18	DN17539-R	CACACGCTTCTCATACAATT	20	190
DN20046-F	ATCATCTTCCTCCTCTTCCT	20	DN20046-R	TGCTGGTTCCTTCATAATCT	20	197
DN11176-F	TGTTATGTCGCTGTGAGTT	19	DN11176-R	GAATCCTCTGTTGCTAATCG	20	193
DN22577-F	GGAGGAACTAAGTGATGAGAT	21	DN22577-R	AACAGTAACAGTGACGAGAT	20	169
DN7316-F	ATAACAGAGGACGGCATTG	19	DN7316-R	TGGCGATTCAAGCACATT	18	191
DN29156-F	CAGGTGGAGAAGCATAGG	18	DN29156-R	ACGACTGAACATCTGAGAA	19	188
DN15094-F	AACATACCGCTTCATATTGG	20	DN15094-R	ATCATTCGCTGTAGGAGAC	19	197
DN33670-F	GCCTAACTGAATCCTTGCTA	20	DN33670-R	GCTCATACCTCTTACCGATAT	21	145
DN17132-F	GGTTCACTTAGCCACGATT	19	DN17132-R	ATCCGAGAGCCTTGACAG	18	166
DN11424-F	CTCAATTCTCATTCCACCATC	21	DN11424-R	TACATCTCCAACAACCTCAG	20	172
DN13837-F	CATTGCTTGCTTGCTCTC	18	DN13837-R	TGACCTTCGGCTAGACAT	18	160
18SrRNA-F	GGTCCAACTACGAGCTTTTTAACT	24	18SrRNA-R	TTTTAGCCCGTTGCTCTGATG	21	180
